# Interleukin‐1α Mediates Pancreatic Fibroblast Activation, Regulates Immune Cell Recruitment and Fibrosis in Acute and Chronic Pancreatitis

**DOI:** 10.1002/advs.77558

**Published:** 2026-09-03

**Authors:** Hala Mazloum, Shenja Buchholz, Hanna Schifter, Sabine Ameling, Leif Steil, Ben Lewin Becker, Elke Hammer, Georg Homuth, Uwe Völker, Barbara M. Bröker, Lukas Zierke, Frank Ulrich Weiss, Juliane Glaubitz, Matthias Sendler

**Affiliations:** ^1^ Department of Medicine A University Medicine Greifswald Greifswald Germany; ^2^ Interfaculty Institute for Genetics and Functional Genomics University Medicine Greifswald Greifswald Germany; ^3^ German Centre for Cardiovascular Research (DZHK) Partner Site Greifswald Greifswald Germany; ^4^ Institute of Immunology University Medicine Greifswald Greifswald Germany

**Keywords:** acute pancreatitis, Anakinra, chronic pancreatitis, fibroblasts, fibrosis, Interleukin 1 alpha

## Abstract

Pancreatitis is a life‐threatening inflammatory disease of the pancreas. The cytokine interleukin‐1α has been demonstrated to act as an alarmin released by necrotic cells. In the present study, we investigated the influence of IL‐1α on the immune response during acute and chronic pancreatitis. Following tissue injury, pancreatic acinar cells released IL‐1α, which activates tissue‐resident fibroblasts to differentiate toward a pro‐inflammatory phenotype. By secreting chemokines and cytokines such as CXCL5, CCL2, and IL‐6, these fibroblasts recruit immune cells to the pancreas. The absence of IL‐1α reduces disease severity in acute pancreatitis and chemokine release. Furthermore, IL‐1α primes fibroblasts to enhance the production of extracellular matrix‐components by the up‐regulation of pro‐fibrotic receptors such as *Il4ra*, *Il13ra1*, and *Tgfbr3*. Therefore, the deletion of IL‐1α significantly reduced the development of tissue fibrosis. A therapeutic blockade of the IL1R1‐signaling by i.p. administration of the IL‐1‐receptor antagonist Anakinra showed the same effect; the severity of acute pancreatitis and fibrogenesis during chronic pancreatitis were reduced. In conclusion, the crosstalk between necrotic acinar cells and fibroblasts mediated by IL‐1α plays a crucial role in acute inflammation of the pancreas and fibrogenic signaling. Blockade of IL1R1‐signaling by Anakinra is therefore a promising therapeutic intervention for both acute and chronic pancreatitis.

## Graphical abstract

During pancreatitis, IL‐1α is released from necrotic acinar cells. In response to IL‐1α, pancreatic fibroblasts release chemokines and cytokines that regulate the recruitment of immune cells to the damaged organ. Furthermore, IL‐1α primes fibroblasts, resulting in increased tissue fibrosis during chronic pancreatitis. Therefore, blocking the IL‐1R1 signalling pathway by anakinra represents a potential therapeutic approach for the treatment of both acute and chronic pancreatitis.

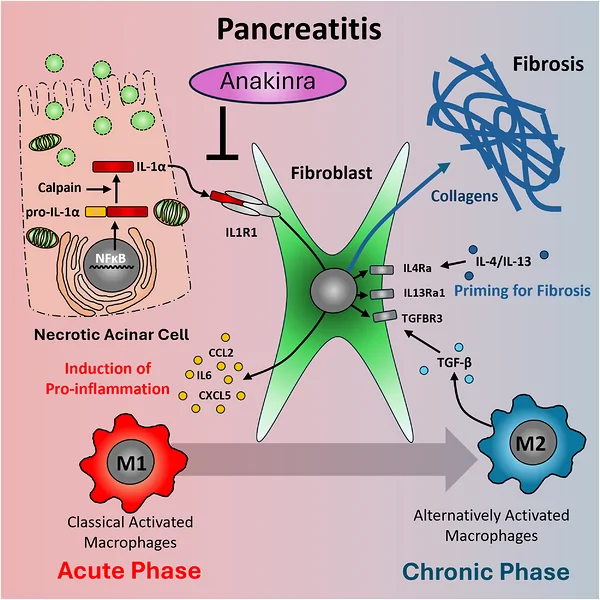

## 1 Introduction

Acute pancreatitis (AP) is the most common non‐malignant gastrointestinal disease leading to hospital admission, with an increasing incidence. In approximately 80% of patients, the disease takes a mild, self‐limiting course. However, in 20% of all cases, severe AP has serious complications such as persistent organ failure and colonization of pancreatic necrosis by commensal intestinal bacteria [1, 2, 3]. One third of the patients with a first episode of AP experience recurrent pancreatitis, which will progress to chronic pancreatitis (CP) in a quarter of these cases [4]. CP is characterized by relapsing episodes of AP and results in a gradual loss of exocrine and endocrine pancreatic tissues, which are replaced by fibrosis over time. Currently, no causal therapy has been developed for either acute or chronic forms of the disease.

The severity of AP as well as the progression of CP are significantly influenced by a complex regulatory network of local and systemic immune responses, in which macrophages play an important role [5]. In the situation of acute acinar cell damage, classically activated macrophages (M1) phagocytose necrotic cell debris and release pro‐inflammatory mediators such as IL‐6, TNF‐α, or IL‐1β, which activate and recruit further immune cells to the pancreas and increase the local damage [6, 7, 8]. Following the clearance of necrotic areas, polarization of the macrophages changes to an alternatively activated phenotype (M2), which is generally associated with wound healing, fibrosis, and regeneration [9, 10, 11]. M2‐macrophages release growth factors like TGF‐β that drive pancreatic fibrosis by activating pancreatic stellate cells and fibroblasts to produce extracellular matrix proteins such as collagens [12].

The communication between the various cell types involves signal transmission by cytokines and chemokines. Interleukin 1α, along with IL‐1β and IL‐18 are classified as a member of the IL‐1 cytokine family, all sharing the property of requiring proteolytic activation for optimal function [13, 14]. While IL‐1β and IL‐18 are mainly processed and released by macrophages via activation of the inflammasome complex as well as caspase‐1 activation, IL‐1α is produced ubiquitously in nearly all cell types. IL‐1α is considered an alarmin and is released during necrotic cell death to initiate an immune response [15]. Both IL‐1α and IL‐1β bind to the IL‐1 receptor type 1 (IL1R1) and induce activation of the transcription factor NFκB via the MyD88/IRAK signaling pathway. While IL‐1β and the activation of the inflammasome complex have been well studied in the context of pancreatitis [7, 16, 17], there is limited research on the role of IL‐1α.

Here, using *Il1a‐/‐* mice, we have analyzed how the immune response during AP is influenced by IL‐1α. Our study reveals that IL‐1α activates tissue‐resident pancreatic fibroblasts, which in turn differentiate into a pro‐inflammatory phenotype and initiate the recruitment of immune cells via release of chemokines. Consequently, it can be concluded that tissue‐resident fibroblasts function as cellular damage sensors, able to trigger the immune response in acute pancreatitis. Furthermore, we show that IL‐1α has a priming effect on fibroblasts, which promotes fibrogenesis during the development of CP. We show that blockade of the IL1R1 signaling pathway may represent a promising strategy for the treatment of pancreatitis.

## 2 Results

### 2.1 Damaged Acinar Cells Release Interleukin 1‐Alpha But Not Interleukin 1‐Beta

**FIGURE 1:**
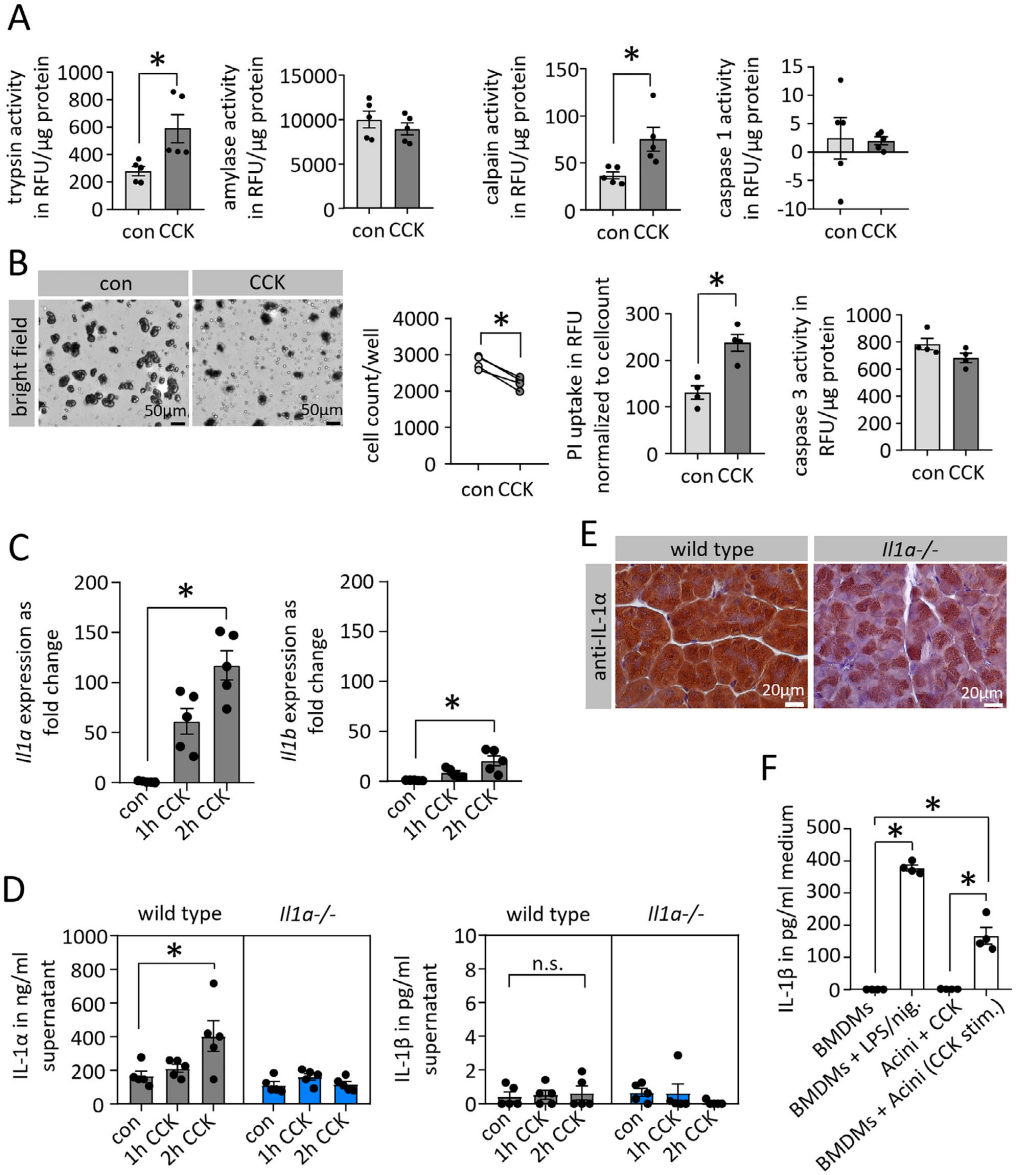
Damaged acinar cells release Interleukin 1 alpha but not Interleukin 1 beta. (A) Pancreatic acinar cells were isolated from wild type mice and stimulated with 0.001 mm CCK for 60 min. Activity of trypsin, amylase, calpain, and caspase 1 was measured in cell homogenate (*n* = 5). (B) Determination of acinar cell death by measurement of cell count and propidium iodide (PI) uptake in acinar cells 60 min after stimulation with 0.001 mm CCK (*n* = 4). Caspase 3 activity was determined 60 min after CCK stimulation in cell homogenate (*n* = 4). (C) Reverse Transcription‐quantitative PCR (RT‐qPCR)‐analysis of *Il1a* and *Il1b* was performed from isolated pancreatic acinar cells following stimulation with 0.001 mm CCK for 1 h and 2 h (*n* = 5). (D) The release of IL‐1α and IL‐1β into medium was measured in acinar cells isolated from wild type and *Il1a‐/‐* mice, after stimulation with 0.001 mm CCK for 1 h and 2 h (*n* = 5). Differences were tested for statistical significance by unpaired student's *t*‐test for independent samples, and significance levels of *p* < 0.05 are marked by an asterisk. (E) Immunohistochemical labeling of IL‐1α on healthy pancreas tissue sections of wild‐type and IL‐1α deficient mice (*Il1a‐/‐*). (F) IL‐1β release was measured in the supernatant of bone marrow‐derived macrophages (BMDMs) after stimulation with LPS and nigericin. Furthermore, IL‐1β release was measured in the supernatant of CCK‐stimulated acinar cells and BMDMs co‐incubated with these damaged acinar cells (*n* = 4). Differences were tested for statistical significance by one‐way ANOVA corrected for multiple testing by Tukey's Post‐hoc‐test, significance levels of p < 0.05 are marked by an asterisk.

A hallmark of acute pancreatitis is the proteolytic self‐digestion of the pancreas by its own proteases. The activation of trypsinogen to trypsin marks the disease onset and results in necrotic cell death of pancreatic acinar cells. In response to high concentrations of cholecystokinin (0.001 mm CCK), acinar cells exhibited a significant activation of trypsinogen to trypsin, while amylase was already present in an active form in unstimulated cells. In addition to the elevated activity of digestive proteases, CCK stimulation also increased the activity of calpain, whereas the IL‐1β converting enzyme caspase‐1 showed no activity in the presence or absence of CCK stimulation (Figure 1A). The intracellular activation of digestive enzymes results in necrotic cell death [18, 19] of acinar cells, as illustrated by significantly elevated PI uptake after CCK stimulation, whereas caspase‐3 activity, a marker of apoptotic cell death, showed no significant induction (Figure 1B). IL‐1α as well as IL‐1β need to undergo proteolytic processing during maturation. The activation of pro‐IL‐1β is caspase‐1 dependent (a part of the inflammasome complex), whereas processing of IL‐1α can be facilitated by a variety of proteases, including calpain. In addition to the activation of trypsin, the initiation of pro‐inflammatory NFκB signaling is a hallmark of the onset of the pancreatitis‐induced immune response [20]. NFκB regulates the transcription of genes encoding pro‐inflammatory mediators and cytokines, including IL‐1α and IL‐1β [13, 21]. IL‐1α is expressed in nearly all cell types, including pancreatic acinar cells, where it functions as an alarmin to signal cell damage and initiate immune responses (Figure 1C) [13]. Following CCK stimulation of isolated pancreatic acinar cells from C57Bl/6‐J mice, the expression of *Il1a* and *Il1b* was elevated (Figure 1C), with a more substantial increase in *Il1a* mRNA levels. In the next step, we tested whether IL‐1α or IL‐1β are released from acinar cells. The measurement of IL‐1α in the culture medium of CCK‐stimulated acinar cells demonstrated a significant increase in IL‐1α 2 h after stimulation. In contrast, no IL‐1β was detectable (Figure 1D). These results were consistent with our previous activity measurements of calpain and caspase‐1. In contrast to the marked increase in IL‐1α converting protease calpain, a caspase‐1 activity remained undetectable. In IL‐1α deficient mice, neither expression nor release of the cytokine was detected (Figure 1D,E). Furthermore, we compared the release of IL‐1β from bone marrow‐derived macrophages (BMDMs) and isolated acinar cells. We observed a significant release of mature IL‐1β from BMDMs after co‐incubation with CCK‐stimulated acini, whereas CCK‐stimulated acini alone did not show any IL‐1β release into the medium 6 h after stimulation with CCK (Figure 1F). These experiments demonstrate that only IL‐1α but not IL‐1β is released by pancreatic acinar cells and may affect disease severity.

### 2.2 IL‐1α Exerts Its Effects on Fibroblasts, With No Effect on Macrophages

**FIGURE 2:**
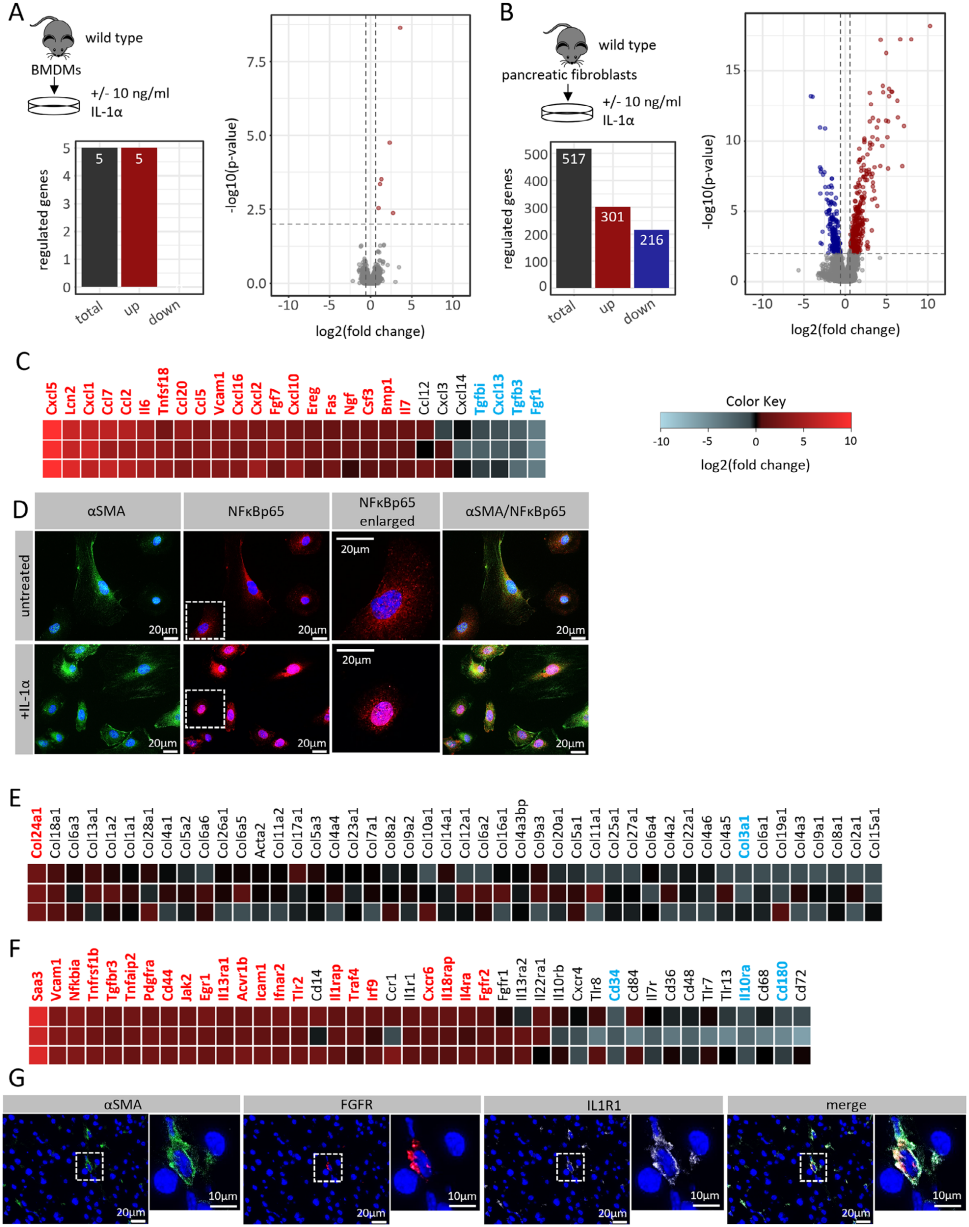
IL‐1α exerts its effects on fibroblasts, with no effect on macrophages. (A) Bone marrow‐derived macrophages (BMDMs) were isolated from wild‐type mice and stimulated with 10 ng/mL recombinant murine IL‐1α for 24 h. Microarray‐based transcriptome analysis using Clariom S mouse arrays was performed using RNA prepared from these BMDMs. Bar graph and Volcano plot illustrate the differentially expressed genes in BMDMs (*n* = 3). (B) Fibroblasts were isolated from the pancreas of wild‐type mice and stimulated with 10 ng/mL IL‐1α for 24 h. Transcriptome analysis was performed using Clariom S mouse arrays, bar graph and Volcano plot illustrate the differentially expressed genes in fibroblasts (*n* = 3). (C) Heatmap illustration of pro‐inflammatory genes exhibiting increased or decreased transcript levels in fibroblasts after IL‐1α treatment (significantly increased: red; significantly decreased: blue). (D) Immunofluorescence labeling of αSMA and NFκBp65 in isolated murine pancreatic fibroblasts ± 10 ng/mL IL‐1α. (E) A heatmap based on transcriptome analysis data illustrates the absence of a pro‐fibrotic profile in IL‐1α‐stimulated fibroblasts. (F) Another heatmap shows a selection of genes encoding receptors and activation markers exhibiting increased or decreased transcript levels in IL‐1α‐treated pancreatic fibroblasts (significantly increased: red; significantly decreased: blue). (G) Images of fluorescent labeling of αSMA^+^, FGFR^+^, and IL1R1^+^ fibroblasts in sections of murine healthy pancreas. Significance was tested by using analysis of variance (ANOVA) with e‐Bayesian correction. Statistically significant differential gene expression in terms of transcript levels was defined as a fold change of > 1.5 fold between the compared conditions and a FDR < 0.05.

Next, our aim was to determine specific cell populations that exhibit a response to IL‐1α. In a first experimental setup, bone marrow‐derived macrophages (BMDMs) from C57Bl/6‐J mice were isolated and subsequently stimulated with 10 ng/mL IL‐1α for 24 h. Surprisingly, BMDMs barely responded to IL‐1α. Microarray‐based transcriptome analysis revealed a modest but statistically significant upregulation of only five genes at the mRNA level (Figure 2A). In contrast to BMDMs, a large number of genes were regulated by IL‐1α in fibroblasts, which had been isolated from the pancreas of C57Bl/6‐J mice. (Figure 2B). The transcriptome analysis revealed a pronounced upregulation of genes indicating a pro‐inflammatory response, particularly with regard to the chemokines *Cxcl5*, *Cxcl1*, *Ccl7*, *Ccl2*, *Cxcl16*, *Cxcl2*, and *Cxcl10*, as well as the cytokine *Il6* (Figure 2C). Ingenuity Pathway Analysis (IPA) software was utilized to summarize the most affected pathways in fibroblasts after IL‐1α stimulation. (Figure S1A). Pro‐inflammatory pathways such as ´Acute Phase Response Signaling´ were significantly activated, whereas anti‐inflammatory pathways such as ´IL‐10 Signaling´ were suppressed. Upstream regulators, including the pro‐inflammatory transcription factors NFκB and RelA, which activate transcription of genes encoding pro‐inflammatory mediators, exhibited an activation pattern (Figure S1B). Fluorescent labeling of NFκBp65 demonstrated a distinct nuclear redistribution of the transcription factor in fibroblasts in response to IL‐1α (Figure 2D). Contrary to our expectations, the results of the transcriptome analysis revealed no pro‐fibrotic global gene expression signature (Figure 2E). IL‐1α, also known as fibroblast‐activating factor [22], did not induce transcription of genes encoding extracellular matrix proteins, including collagens (*Col3a1*, *Col1a1)* or alpha‐smooth muscle actin (*Acta2*), the classical activation marker of pancreatic stellate cells. However, pathway analyses revealed induction of the ‘hepatic fibrosis/hepatic stellate cell activation’ signaling pathway (Figure S1A). In addition to the activation of fibroblasts toward a pro‐inflammatory phenotype, we observed increased mRNA levels of various receptors, including the TGF‐receptor 3, the IL‐4 receptor, and the IL‐13 receptor (Figure 2F), which suggests that the fibroblasts were primed for pro‐fibrotic signals such as transforming growth factor‐beta (TGF‐β). Besides these gene expression changes, we observed increased cell proliferation in response to IL‐1α (Figure S1C). Proteome analysis of fibroblasts and BMDMs stimulated with 10 ng/mL IL‐1α confirmed the results of the transcriptome analyses and demonstrated significantly increased levels of activation markers CD44 and TGF‐β receptor TGBR3 in fibroblasts, but no significant differences in the protein composition of BMDMs in response to IL‐1α. The non‐fibrotic phenotype of IL‐1α‐stimulated fibroblasts is corroborated by a significant reduction of the protein levels of collagen 3A1 (CO3A1) and desmoplakin (DESP) (Figure S2A,B). Quiescent pancreatic fibroblasts are a component of the cellular landscape in the healthy pancreas. These αSMA/FGRF producing cells also carry the IL1R1 on their surface, enabling them to respond to IL‐1α (Figure 2G). In summary, the experimental results indicated that fibroblasts, in contrast to macrophages, respond to IL‐1α and differentiate into a pro‐inflammatory phenotype. Upregulation of receptor gene expression primes the cells for additional or subsequent pro‐fibrotic signals.

### 2.3 Deficiency of IL‐1α Ameliorate Disease Severity of Acute Pancreatitis in Mice

**FIGURE 3:**
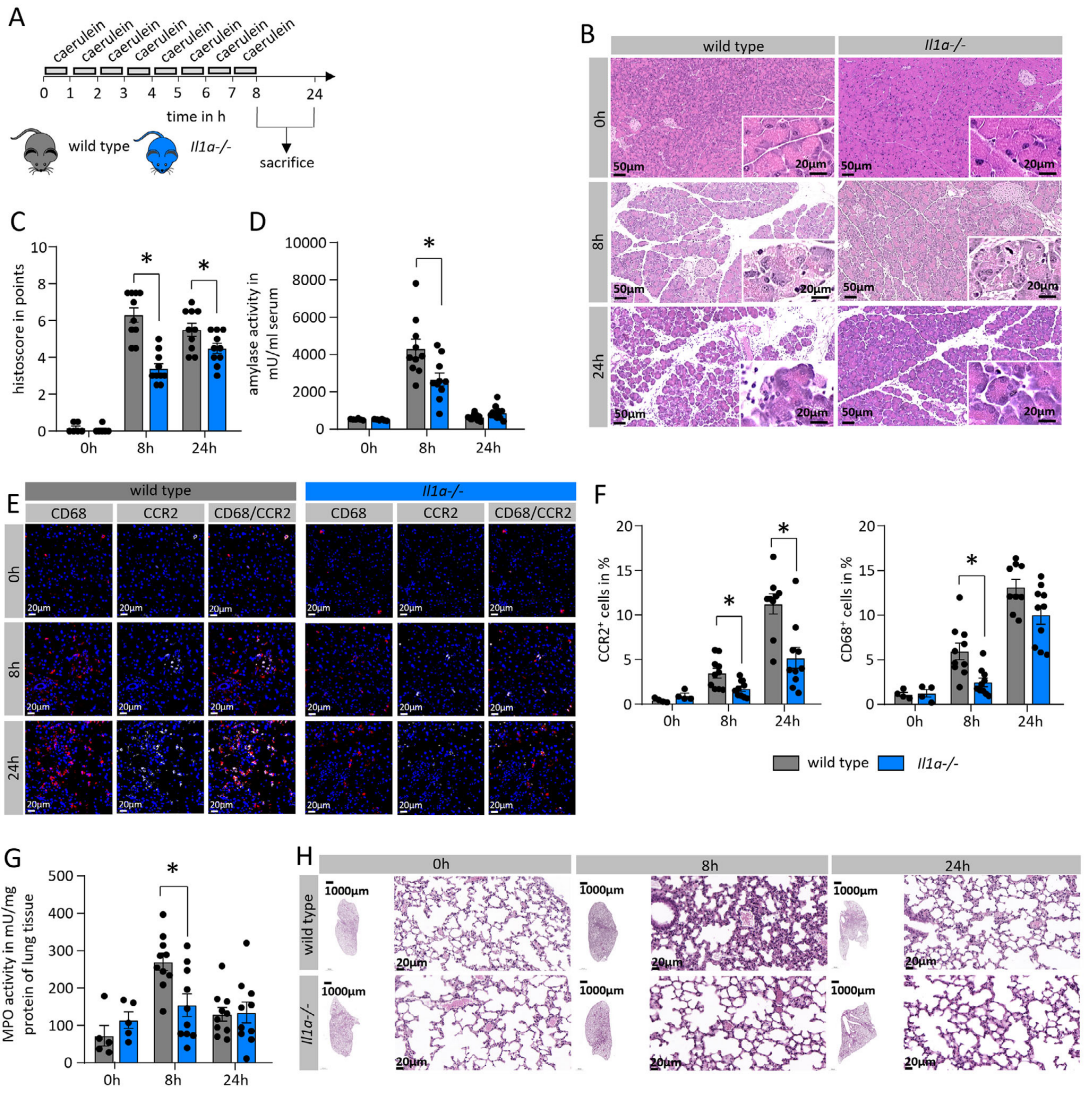
Deficiency of IL‐1α ameliorates the disease severity of acute pancreatitis in mice. (A) Acute pancreatitis was induced by hourly i.p. injections of caerulein in wild‐type and *Il1a‐/‐* mice as shown schematically (*n* = 10 for 8 and 24 h each strain). (B) H&E staining of pancreatic tissue sections. (C) Evaluation of H&E histology by histoscore. (D) Measurement of amylase activity in serum of mice. (E) Immunofluorescent labeling of CD68 and CCR2 in pancreatic tissue sections. (F) Quantification of CD68^+^ and CCR2^+^ cells in pancreatic tissue. (G) Measurement of myeloperoxidase (MPO) activity in lung tissue homogenates. (H) H&E staining of lung tissue sections. Differences were tested for statistical significance by unpaired Student's *t*‐test for independent samples, and significance levels of p < 0.05 are marked by an asterisk.

To address the question if IL‐1α affects disease severity in a mouse model of AP, the disease was induced by hourly i.p. injections of caerulein in wild‐type and *Il1a‐/‐* mice (Figure 3A). The assessment of the pancreatic damage was based on H&E staining, which revealed less tissue injury in *Il1a‐/‐* mice than in wild‐type animals (Figure 1B). In the *Il1a‐/‐* mice, the number of necrotic acinar cells was significantly reduced, and there were less infiltrating immune cells 8 and 24 h after the onset of AP (Figure 1C and Figure S3A–C). Activity measurements of serum amylase and lipase were consistent with the histological findings and were significantly reduced 8 h after the induction of AP (Figure 3D and Figure S3D). The release of the alarmin IL‐1α from necrotic cells results in the induction of tissue inflammation, so it was not surprising that we observed decreased numbers of CD68^+^ macrophages and CCR2^+^ infiltrating cells in the pancreas of the IL‐1α‐deficient mice (Figure 3E,F). Additionally, the systemic inflammation was found to be considerably diminished in the *Il1a‐/‐* mice in comparison to the wild‐type animals. This observation is substantiated by a reduction in myeloperoxidase activity in lung tissue homogenates and by the preserved lung histology (Figure 3G,H). Our observation of lower numbers of infiltrating immune cells in *Il1a*‐/‐ mice undergoing experimental pancreatitis provides an explanation for the reduced disease severity compared to wild‐type animals. Our findings suggest crosstalk between tissue‐resident fibroblasts and macrophages/monocytes during AP.

### 2.4 IL‐1α Induces Crosstalk Between Pancreatic Fibroblast and Immune Cells

**FIGURE 4:**
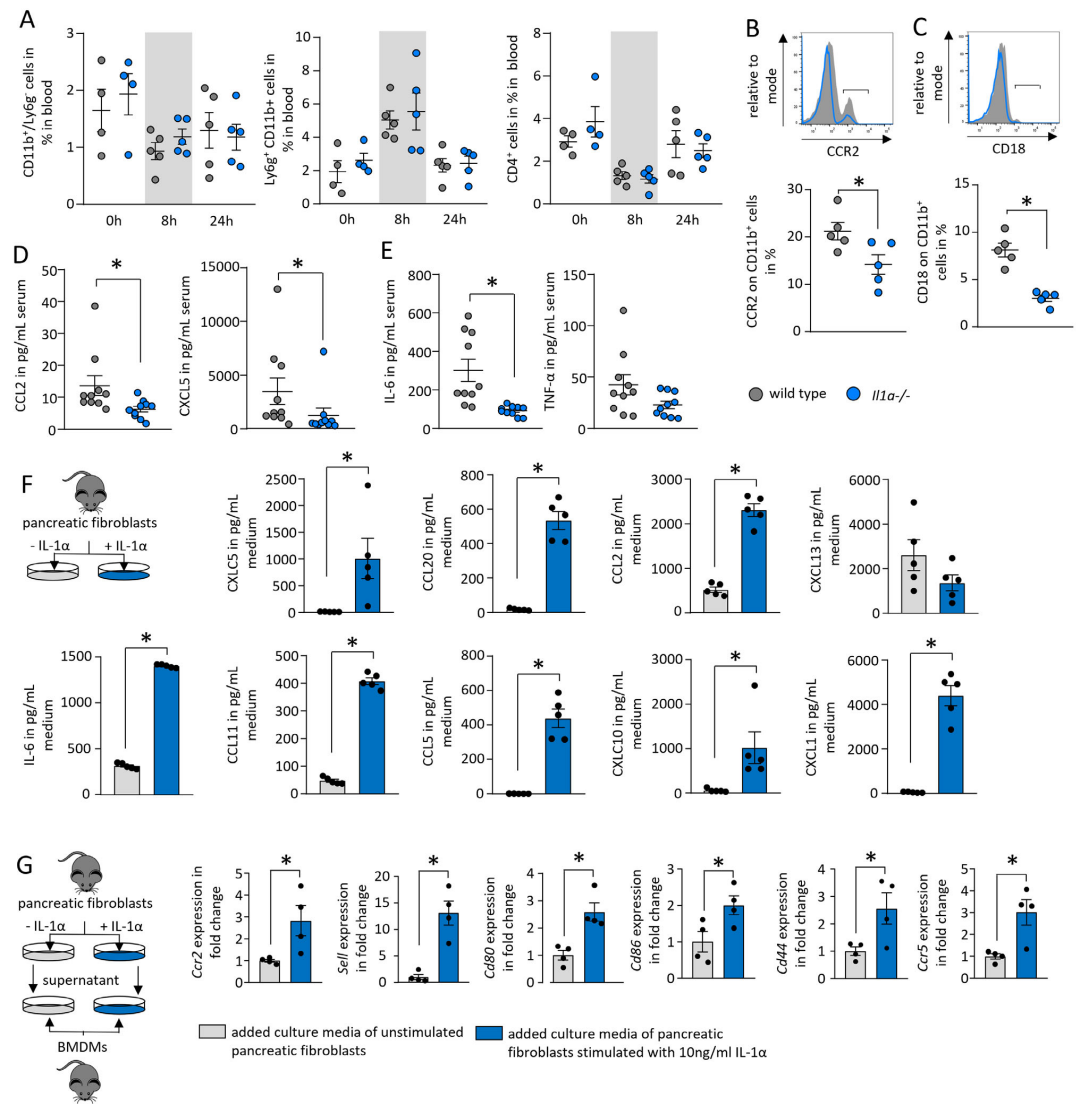
IL‐1α activates crosstalk between pancreatic fibroblast and immune cells. (A) Flow cytometry analysis of blood leukocytes, isolated from wild‐type and *Il1a‐/‐* mice at eight and 24 h after onset of AP. The population of CD11b^+^/Ly6g^−^ reflects monocytes; CD11b^+^/Ly6g^+^ cells were identified as neutrophils, whereas CD4^+^ cells marked the population of T helper cells (*n* = 4‐5). (B, C) Histogram and dot plot illustrate the expression of CCR2 and CD18 on the surface of CD11b^+^ cells in blood, 8 h after onset of pancreatitis (*n* = 5). (D) Measurement of the chemokines CCL2 and CXCL5 in serum of mice 8 h after onset of disease (*n* = 10). (E) Measurement of the cytokines IL‐6 and TNF‐α in serum of mice 8 h after induction of pancreatitis (*n* = 10). (F) Measurement of chemokines and cytokines in the culture media of isolated pancreatic fibroblasts which were stimulated with 10 ng/mL IL‐1α for 24 h (*n* = 5). (G) The supernatant of isolated pancreatic fibroblasts ± IL‐1α stimulation for 24 h was added to BMDMs for an additional 24 h. RNA was isolated from the BMDMs culture, and expression of *Ccr2*, *Sell*, *Cd80*, *Cd86*, *Cd44*, and *Ccr5* was analysed by RT‐qPCR (*n* = 4). Differences were tested for statistical significance by unpaired student's *t*‐test for independent samples, and significance levels of p < 0.05 are marked by an asterisk.

Subsequently, we examined the underlying mechanism in detail. Monocytes and neutrophils migrate from the blood to the pancreas during the acute phase of pancreatitis, but no differences were detected between wild‐type and *Il1α‐/‐* mouse strains concerning the composition of circulating immune cells. CD11b^+^/LY6G^−^ monocytes, neutrophil granulocytes CD11b^+^/LY6G^+^ and CD4^+^ T helper cells were present in comparable numbers (Figure 4A and Figure S4A,B). Since the absence of IL‐1α did not affect the number of circulating immune cells, we next investigated cell migration. At 8 h after pancreatitis induction, the levels of chemokine receptor CCR2 and the β2 integrin CD18 on the surface of circulating CD11b^+^ cells in *Il1a‐/‐* mice were significantly reduced (Figure 4B,C). The gene expression of chemokine receptors and integrins, as well as their activation, is regulated by chemokines [23, 24]. We observed several chemokine‐encoding genes to be transcriptionally upregulated in fibroblasts following stimulation with IL‐1α (Figure 2C). In the absence of IL‐1α we detected a significant decrease in serum concentrations of CCL2 and CXCL5 8 h after onset of disease in *Il1a‐/‐* animals (Figure 4D). Furthermore, the serum concentration of IL‐6, but not TNF‐α, was significantly reduced in IL‐1α‐deficient animals, which is consistent with the results of the transcriptome analyses of fibroblasts (Figure 2C and Figure 4E). We reasoned that IL‐1α may act on pancreatic fibroblasts and stimulated isolated fibroblasts from the pancreas with IL‐1α to determine the release of specific chemokines into the culture medium. Following stimulation of the fibroblasts with 10 ng/mL IL‐1α, the levels of CCL20, CCL2, CCL11, CCL5, CXCL10 and CCL1, as well as of IL‐6 were significantly increased (Figure 4F). To investigate the crosstalk between pancreatic fibroblasts and macrophages in more detail, we isolated BMDMs from wild‐type mice and stimulated them with conditioned culture medium from pancreatic fibroblasts after IL‐1α stimulation. While IL‐1α did not affect these cells, the conditioned medium of IL‐1α ‐stimulated fibroblasts caused a significant transcriptional up‐regulation of activation markers *Cd80*, *Cd86*, and *Cd44* in the macrophages. Additionally, increased mRNA levels of the chemokine receptors CCR2 and CCR5, as well as of the cell adhesion molecule L‐selectin encoded by *Sell* were observed (Figure 4G). These findings demonstrate a critical impact of IL‐1α‐activated pancreatic fibroblasts on monocyte migration and activation and reveal their pivotal role in the initiation of the immune response.

### 2.5 Therapeutic Blockage of the IL1R1 Signaling Pathway by Anakinra Reduces Disease Severity in a Mouse Model of AP

**FIGURE 5:**
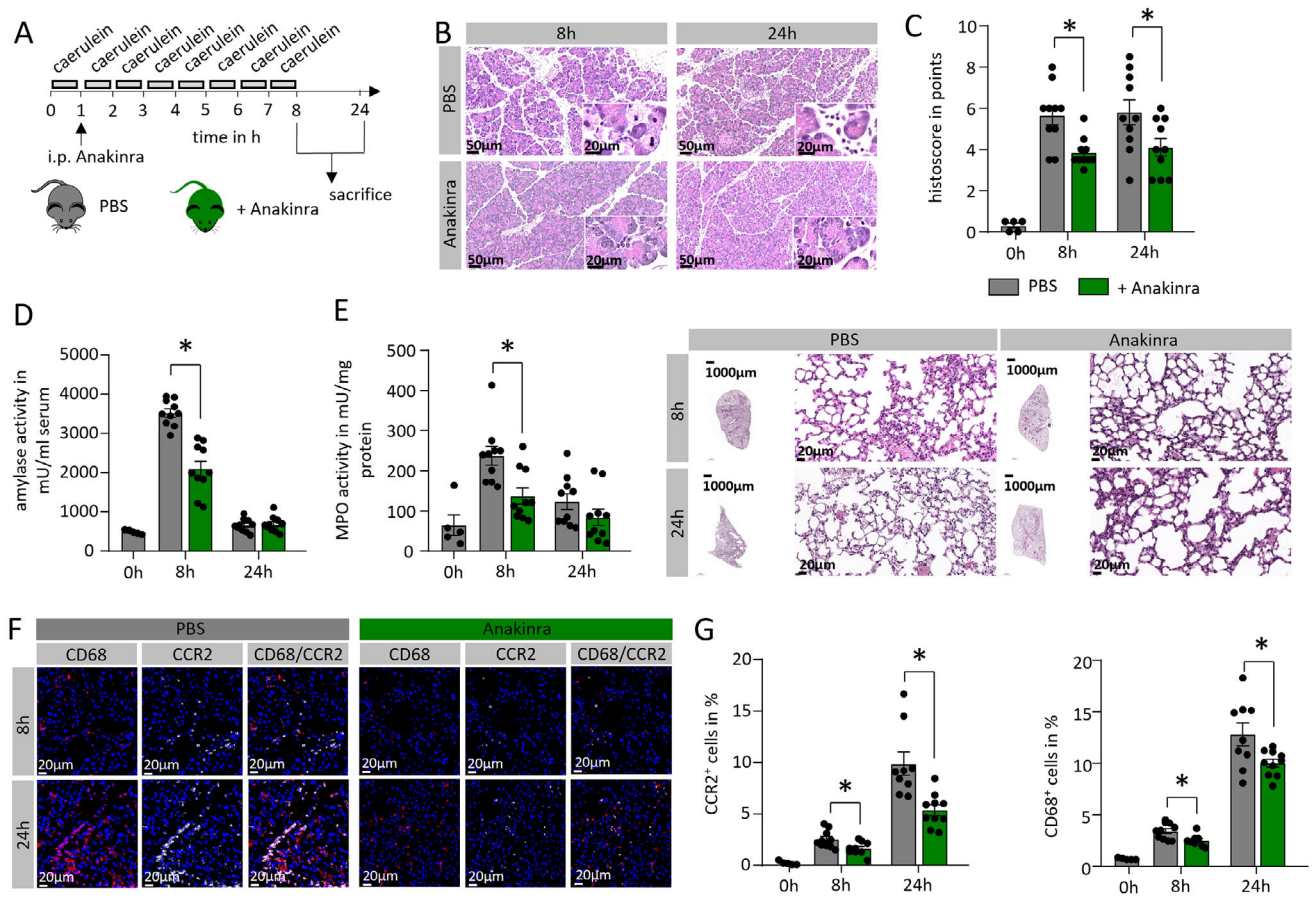
Therapeutic blockage of the IL1R1 signaling pathway by Anakinra reduces the disease severity in a mouse model of AP (A) Acute pancreatitis was induced by hourly i.p. injections of caerulein in wild‐type mice. The treatment group received a single application of 10 mg/kg body weight Anakinra 1 h after the first caerulein injection, whereas the control group received PBS (*n* = 10 for 8 and 24 h). (B) H&E histology of pancreatic tissue sections. (C) Evaluation of H&E histology by histoscore. (D) Measurement of amylase activity in serum of mice. (E) Measurement of myeloperoxidase (MPO) activity in lung tissue homogenates and H&E staining of lung tissue sections. (F) Immunofluorescent labeling of CD68 and CCR2 in pancreatic tissue sections. (G) Quantification of CD68^+^ and CCR2^+^ cells in pancreatic tissue. Differences were tested for statistical significance by unpaired student's *t*‐test for independent samples, and significance levels of p<0.05 are marked by an asterisk.

To date, no causal therapy for the treatment of acute pancreatitis has been developed, and we were curious whether an inhibition of the IL1R1 signaling pathway could be a therapeutic option. Therefore, we attempted to phenocopy IL‐1α deficiency by a blockade of the IL‐1R1 with the recombinant IL1 receptor antagonist Anakinra. AP was induced by caerulein injection in C57Bl/6‐J wild‐type mice. One hour after the initial caerulein injection, the treatment group received one intraperitoneal dose of Anakinra (10 mg/kg/body weight), while the control group received PBS instead (Figure 5A). Pancreas histology revealed a marked decrease in tissue damage in the group treated with Anakinra (Figure 5B,C). At 8 and 24 h after pancreatitis induction, both the numbers of necrotic acinar cells and those of infiltrating immune cells were significantly lower (Figure S5A–C). Additionally, markers of pancreatic damage, such as serum amylase and lipase activities, were significantly reduced in the Anakinra‐treated group (Figure 5D and Figure S5D). Like the *Il1a* knockout Anakinra treatment significantly reduced the systemic immune response, which is reflected by a decreased MPO activity in lung tissue 8 h after pancreatitis onset (Figure 5E). In the same way, Anakinra treatment caused a significant reduction of the local immune response, exhibited by reduced numbers of CD68^+^ pancreatic macrophages and CCR2^+^ infiltrating immune cells in Anakinra‐treated mice (Figure 5F,G). The analogous results in the Anakinra treatment model and in the IL‐1α genetic deletion model identify the IL‐1R1 signaling pathway as a therapeutic target for the treatment of AP.

### 2.6 IL‐1α Primes Fibroblasts and Promotes Fibrogenesis Indirectly

**FIGURE 6:**
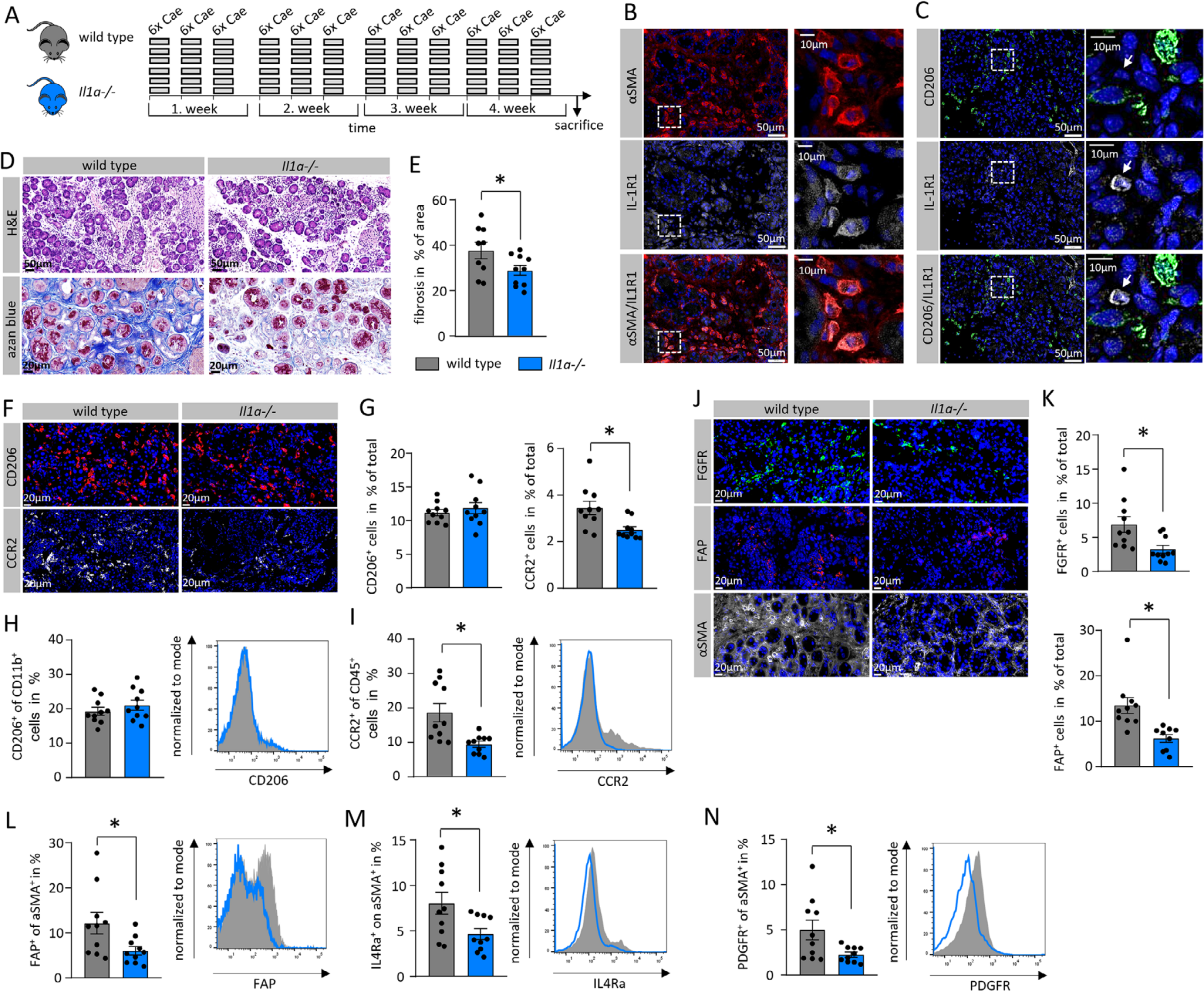
*IL‐1α primes fibroblasts and promotes fibrogenesis indirectly*. (A) Chronic pancreatitis was induced by repetitive caerulein injections over 4 weeks in wild type and *Il1a‐/‐* mice (*n* = 10 each strain). (B) Immunofluorescent labeling of IL1R1 (white) and αSMA (red) positive fibroblasts in CP tissue sections of wild type mice. (C) Immunofluorescent labeling of IL1R1 (white) and CD206 (green) positive macrophages in CP tissue of wild type mice. (D) H&E histology and azan blue staining of CP tissue sections illustrate the tissue architecture and fibrosis. (E) Quantification of azan blue fibrotic area in CP tissue. (F) Immunofluorescent labeling of CD206^+^ macrophages and CCR2^+^ immune cells in CP tissue sections of mice. (G) Quantification of immunofluorescent‐labeled CD206^+^ and CCR2^+^ cells in CP tissue. (H, I) Flow cytometry analysis of isolated cells from CP tissue, (H) CD206^+^ alternatively activated macrophages of the CD11b^+^ cells and (I) CCR2^+^ immune cells of CD45^+^ cells. (J) Immunofluorescent labeling of fibroblasts in CP tissue sections of mice by FGFR (green), FAP (red), and αSMA (white). (K) Quantification of immunofluorescent labeling of FGFR^+^ cells and FAP^+^ cells in CP tissue of wild‐type and *Il1a‐/‐* mice. (L–N) Flow cytometry analysis of FAP, IL4Ra and PDGFR on αSMA^+^ fibroblasts isolated from CP tissue of *Il1a‐/‐* and wild‐type mice. Differences were tested for statistical significance by unpaired Student's *t*‐test for independent samples, and significance levels of p < 0.05 are marked by an asterisk.

Approximately one‐third of patients diagnosed with acute pancreatitis experience recurrent acute pancreatitis, which can further develop into the chronic form of the disease. We employed a mouse model of repetitive caerulein injections over 4 weeks to induce chronic pancreatitis (Figure 6A). In these animals, IL1R1 was detected on αSMA^+^ cells (Figure 6B), but not on CD206^+^ macrophages (Figure 6C). We made the same observation in human CP samples, where we also detected IL1R1 on the surface of αSMA^+^ fibroblasts (Figure S6). We therefore analysed whether the deletion of IL‐1α affects fibrinogenesis in the context of CP. IL‐1α‐deficient mice exhibited significantly decreased tissue fibrosis within the pancreas (Figure 6D,E). CD206^+^ alternatively activated macrophages in the pancreas are considered to be responsible for the induction of fibrosis during CP [10, 11]. However, the numbers of these cells were unchanged in the pancreas of IL‐1α‐deficient mice, whereas a significant reduction of CCR2^+^ infiltrating leukocytes was observed (Figure 6F,G). Flow cytometric analyses of isolated leukocytes from pancreatic tissue confirmed these histologic observations and demonstrated comparable numbers of CD206^+^ alternatively activated macrophages but significantly lower numbers of CCR2^+^ leukocytes in the chronically inflamed pancreas of *Il1a‐/‐* animals (Figure 6H,J). We next investigated whether pancreatic fibroblasts were affected by the deletion of IL‐1α in CP and found a significant decrease in pancreatic FGFR^+^ fibroblasts in the *Il1a‐/‐* mice. The numbers of fibroblast activation protein (FAP)‐positive stromal cells were also significantly diminished. This aligns with the αSMA immunofluorescence labeling which was of lower intensity in *Il1a‐/‐* mice (Figure 6I,K). These findings are consistent with the results of the transcriptome analysis (Figure 2A,B) and indicate that IL‐1α signaling has a much larger impact on fibroblasts than on macrophages. Notably, the IL‐1α‐stimulated pancreatic fibroblasts did not develop a pro‐fibrotic phenotype but showed a significant transcriptional upregulation of genes encoding receptors directly related to fibrosis, including *Tgfbr3*, *Il13ra1*, *Il4ra*, *Acvr1b*, and *Fgfr2* (Figure 2E,F). Proteome analysis revealed that TGBR3 exhibited significant upregulation at the protein level (Figure S2). We next performed a flow cytometry analysis of isolated αSMA^+^ cells from the CP tissue of wild‐type and *Il1a‐/‐* animals. On the surface of the knockout cells the density of FAP, IL‐4Rα and PDGFR was significantly diminished (Figure 6L–N). Together with the transcriptome and proteome data, these findings imply that IL‐1α primes pancreatic fibroblasts by upregulation of their surface receptor setup, thereby increasing sensitivity for fibrogenic stimulation.

**FIGURE 7:**
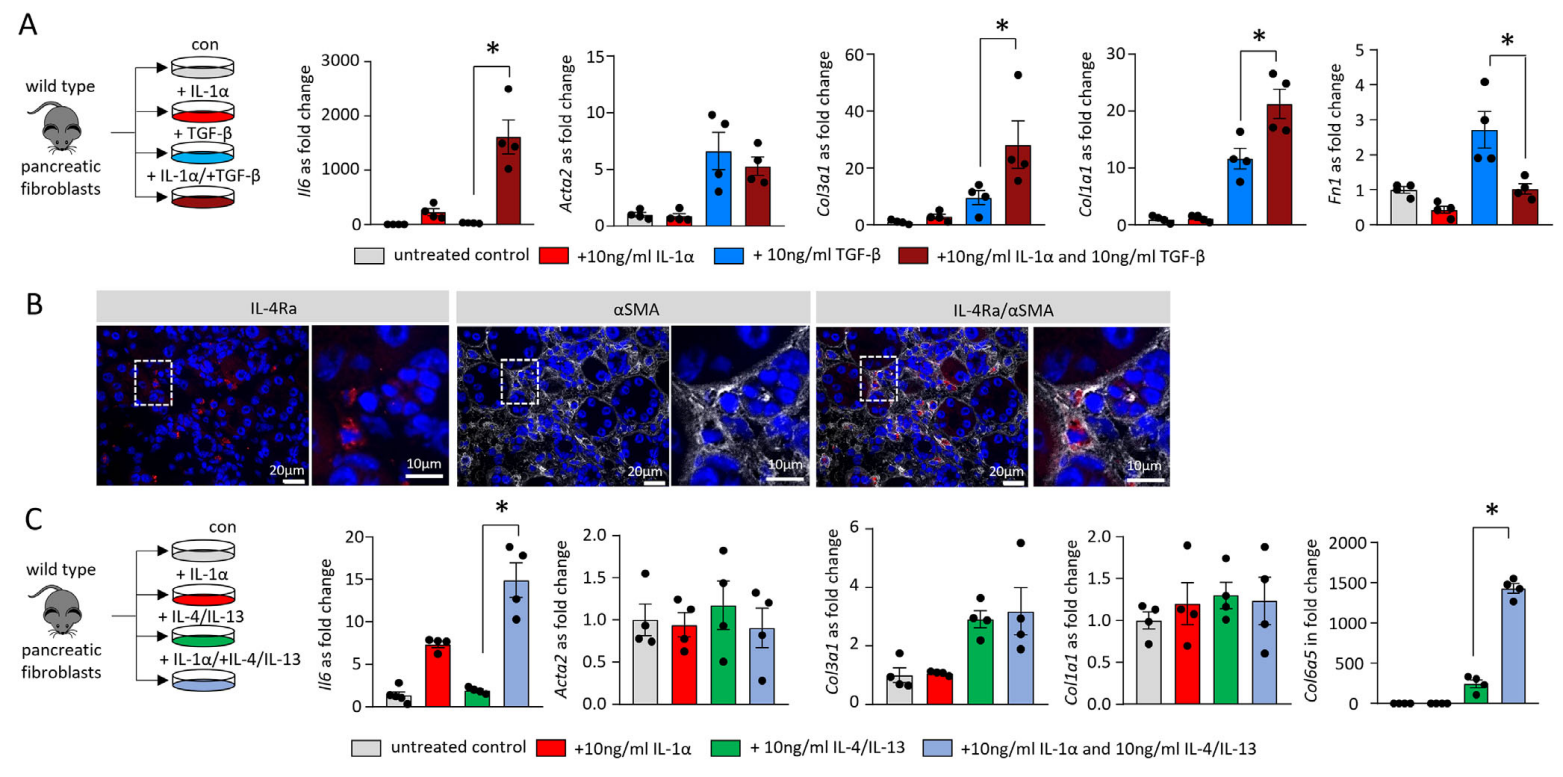
Co‐stimulation of pancreatic fibroblasts with IL‐1α or IL‐4/IL‐13 and TGF‐β enhances collagen mRNA levels. (A) Fibroblasts were isolated from wild type mice and stimulated with 10 ng/mL IL‐1α, 10 ng/mL TGF‐β, or a combination of 10 ng/mL L‐1α and 10 ng/mL TGF‐β for 24 h. A reference was left untreated as controls (con). RNA was isolated from the fibroblast culture, and the expression of *Il6*, *Acta2*, *Col3a1*, *Col1a1*, and *Fn1* were analyzed by RT‐qPCR (*n* = 4 for each condition). (B) Immunofluorescent labeling identified IL‐4Ra (red) and αSMA (white) positive fibroblasts in CP tissue sections of wild‐type mice. (C) Fibroblasts were isolated from wild‐type mice and stimulated with 10 ng/mL IL‐1α, 10 ng/mL IL‐4/IL‐13, or a combination of 10 ng/mL L‐1α and 10 ng/mL IL‐4/IL‐13 for 24 h. A control reference was left untreated(con). RNA was isolated from the fibroblast culture, and the expression of *Il6*, *Acta2*, *Col3a1*, *Col1a1*, and *Col6a5* were analyzed by RT‐qPCR (*n* = 4 for each condition). Differences were tested for statistical significance by one‐way ANOVA followed by Dunnett's multiple comparisons test. Significance levels of p<0.05 are marked by an asterisk.

Our transcriptome and proteome analyses demonstrate a significant upregulation of TGBR3 on fibroblasts following IL‐1α stimulation. CD206^+^ alternatively activated macrophages have been shown to secrete TGF‐β, which triggers the production of extracellular matrix proteins [10]. We therefore co‐stimulated isolated pancreatic fibroblasts with IL‐1α (10 ng/mL) and TGF‐β (10 ng/mL) to see whether the IL‐1α priming affected the pro‐fibrotic TGF‐β signaling. RT‐qPCR analysis revealed that exposure to IL‐1α significantly increased the TGF‐β triggered expression of *Il6*, *Col3a1* and *Col1a1* in fibroblasts, while it did not affect *Acta2* expression and even inhibited the induction of *Fn1*, similar to our previous observations in a mouse model of CP (Figure 7A). In addition to the upregulation of *Tgfbr3*, we also observed a significant upregulation of both *Il13ra1* and *Il4ra* on mRNA level in IL‐1α‐stimulated fibroblasts (Figure 2F). In previous studies, we have shown that the IL‐4/IL‐13/STAT6 signaling pathway directly stimulates the production of extracellular matrix components in fibroblasts in response to IL‐4/IL‐13 [25]. In tissue sections from CP mice, we could detect the expression of IL‐4Ra on αSMA^+^ cells within the CP tissue (Figure 7B). To test if IL‐1α priming of pancreatic fibroblasts has an impact on the response of the cells to IL‐4/IL‐13, we co‐stimulated the cells with IL‐1α (10 ng/mL) and IL‐4/IL‐13 (10 ng/mL). Collagens, such as COL6A5, are known to be induced by IL‐4/IL‐13 [25]. The mRNA expression of *Col6a5* and *Il6*, was significantly increased following co‐stimulation with IL‐1α and IL‐4/IL‐13, whereas other collagens such as *Col1a1* or the PSC activation marker *Acta2* were not influenced by stimulation with IL‐4/IL‐13 alone or in combination with IL‐1α (Figure 7C). These results demonstrate a critical role of IL‐1α in the priming of fibroblasts and the initiation of fibrosis during CP.

### 2.7 Blockage of the IL1R1 Signaling Pathway by Anakinra Reduces Fibrosis in a Mouse Model of CP

**FIGURE 8:**
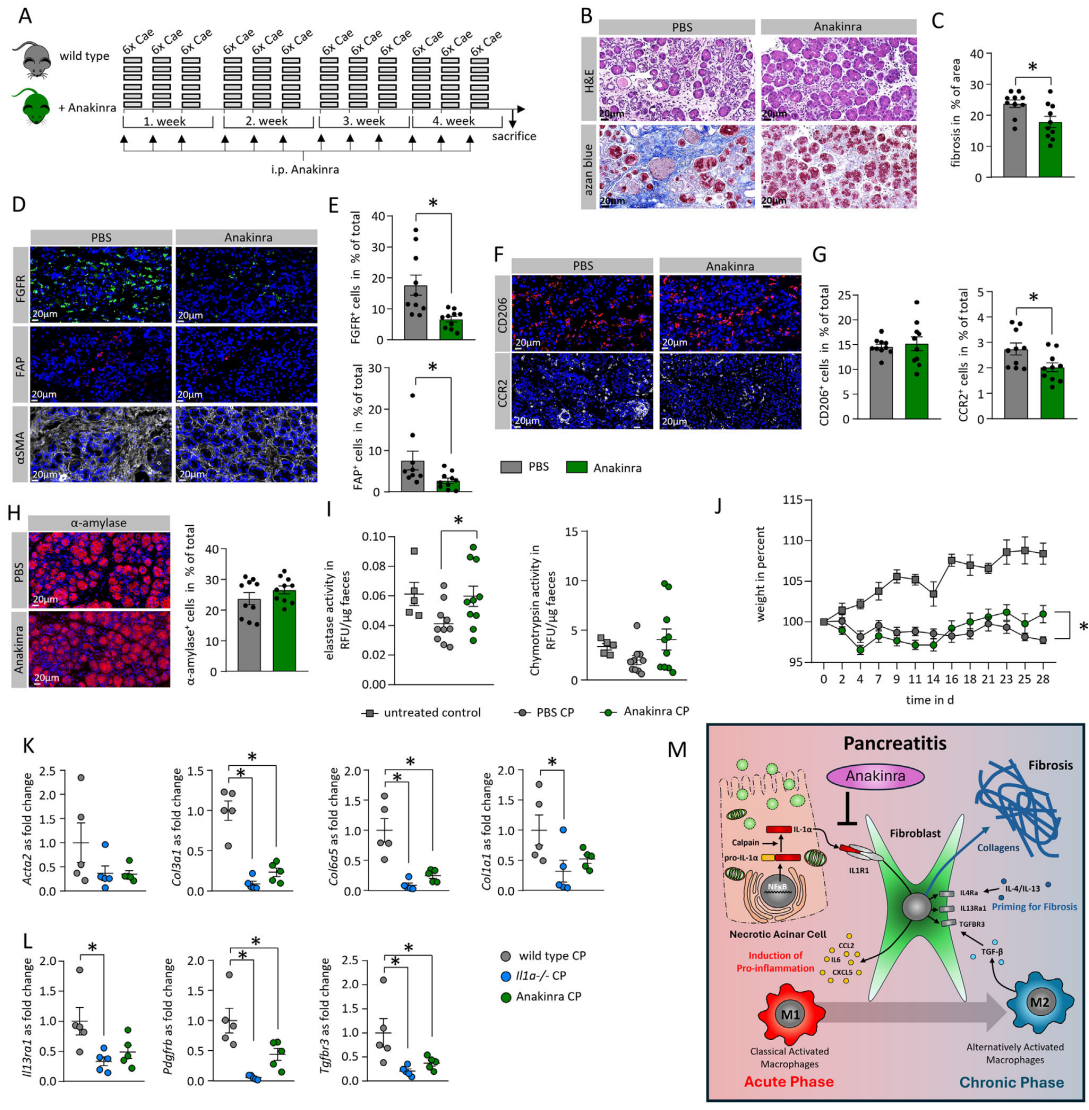
Blockage of the IL1R1 signaling pathway by Anakinra reduces fibrosis in a mouse model of CP. (A) Chronic pancreatitis was induced by repetitive caerulein injections over 4 weeks in wild type mice. The treatment group received a single application of 10 mg/kg bodyweight Anakinra, always before the first caerulein injection, whereas the control group received PBS (*n* = 10 each group). (B) H&E histology and azan blue staining of CP tissue sections illustrate the tissue architecture and fibrosis. (C) Quantification of azan blue fibrotic area in CP tissue. (D) Immunofluorescent labeling of fibroblasts in CP tissue sections of mice by FGFR (green), FAP (red), and αSMA (white). (E) Quantification of immunofluorescent labeling of FGFR^+^ cells and FAP^+^ cells in CP tissue of Anakinra and PBS‐treated mice. (F) Immunofluorescent labeling of CD206^+^ macrophage and CCR2^+^ immune cells in CP tissue sections of mice. (G) Quantification of immunofluorescence‐labeled CD206^+^ and CCR2^+^ cells in CP tissue. Differences were tested for statistical significance by unpaired student's *t*‐test for independent samples, and significance levels of p<0.05 are marked by an asterisk. (H) Labeling and quantification of α‐amylase^+^ cells in CP tissue. (I) Measurement of fecal elastase and chymotrypsin activity in RFU/µg faeces (CP groups *n* = 10, untreated control group *n* = 5). (J) Differences in body weight during induction of CP in mice (CP groups *n* = 10, untreated control group *n* = 5). (K–L) Quantitative RT‐PCR of cells isolated from wild type CP mice, *Il1a‐/‐* CP mice and Anakinra treated CP mice (*n* = 5) for *Acta2*, a marker for activated stellate cells, and the genes *Col3a1*, *Col6a5* and *Col1a1* encoding extracellular matrix proteins and genes encoding pro‐fibrotic receptors such as *Il13ra1*, *Pdgfrb* and *Tgfbr3*. Differences were tested for statistical significance by one way ANOVA followed by Dunnett's multiple comparisons test. Significance levels of p < 0.05 are marked by an asterisk. (M) Graphical abstract summarizes the impact of IL‐1α and its blockage on disease severity of AP as well as fibrinogenesis during CP.

In addition to IL‐1α, also IL‐1β can activate the IL1R1 signaling pathway. However, the determination of serum cytokine levels of IL‐1α and IL‐1β revealed only a significant increase of IL‐1α in CP animals, whereas the serum level of IL‐1β was not increased (Figure S7A). The NLRP3 inflammasome is specifically involved in the maturation and release of IL‐1β during pancreatitis [7, 16]. In contrast to IL‐1α‐deficient mice, a deficiency for NLRP3 showed no beneficial effect on pancreatic fibrogenesis during CP (Figure S7B–F). To evaluate if the IL1R1signaling pathway is a therapeutic option also in CP, we finally investigated whether blocking the IL1R1 signaling pathway with Anakinra has an impact on CP outcome. CP was induced in C57Bl/6‐J mice by caerulein injections as previously described [11]. In addition, mice in the treatment group received one intraperitoneal dose of Anakinra (10 mg/kg/body weight) before the first caerulein injection on each treatment day. The control group received the same volume of PBS (Figure 8A). Consistent with the observations in IL‐1α‐deficient mice, treatment of animals with Anakinra resulted in a significant decrease of pancreatic fibrosis (Figure 8B,C). Furthermore, the number of FGFR^+^ and FAP^+^ cells in the CP tissue of the Anakinra‐treated animals was significantly lower than in that of the PBS control group (Figure 8D,E), and the intensity of the αSMA labeling was lower. Consistent with the observations in the *Il1a‐/‐* animals, the numbers of CD206^+^ alternatively activated macrophages remained unchanged, but those of CCR2^+^ infiltrating cells were reduced (Figure 8F,G). In the next step, we were interested if the treatment with Anakinra also preserved functionally intact pancreatic tissue. We labeled α‐amylase in pancreatic tissue sections and measured the activity of the pancreatic proteases elastase and chymotrypsin in stool samples from the animals. While the number of amylase‐positive cells in the pancreas was marginally elevated in animals treated with Anakinra, a significantly increased elastase activity was observed in the stool samples of Anakinra treated mice (Figure 8H,I). Furthermore, the body weight of animals treated with Anakinra was significantly higher 28 days after the induction of pancreatitis compared to control animals treated with PBS (Figure 8J). Our transcriptome analysis suggested that the pro‐fibrotic effect, mediated by IL‐1α, is induced by the upregulation of various fibrogenic receptors (Figure 2F and Figure 6M,N). Finally, we investigated whether we could also observe this effect in animals treated with Anakinra. Using qRT‐PCR analyses of cells isolated from CP tissue of wild‐type, *Il1a‐/‐* and Anakinra‐treated animals, we examined the mRNA expression of genes encoding extracellular matrix proteins (Figure 8K). We observed a significant reduction in *Col3a1*, *Col6a5* and *Col1a1* in *Il1a‐/‐* animals as well as in mice treated with Anakinra, whilst *Acta2* expression remained unchanged. Also, the expression of the receptors *Il13ra1*, *Pdgfrb* and *Tgfbr3* was significantly reduced in both the *Il1a‐/‐* animals and the mice treated with Anakinra (Figure 8L). These results are in line with the previously observed reduced fibrosis in IL‐1α deficient animals (Figure 6D,E) as well as in animals treated with the IL‐1R1 antagonist Anakinra (Figure 8B,C). In summary, these results suggest that blockage of the IL1R1 receptor pathway could be a promising therapeutic approach in both acute and chronic pancreatitis.

## 3 Discussion

Initial experiments of our current study showed that damaged pancreatic acinar cells release IL‐1α but not IL‐1β. Using *Il1a‐/‐* mice, we demonstrate that IL‐1α directly activates tissue‐resident pancreatic fibroblasts, which, in response to IL‐1α stimulation, differentiate into a pro‐inflammatory phenotype and release chemokines that recruit immune cells to the pancreas. Tissue‐resident fibroblasts thus represent cellular damage sensors that are directly involved in the immune response to pancreatitis.

IL‐1β has been the focus of numerous studies in the context of acute pancreatitis [7, 16, 17]. It is a highly potent cytokine triggering the IL1R1/MyD88 signaling pathway upon binding to target cells, thereby inducing the nuclear translocation of NFκB and the enhancement of a pro‐inflammatory response. However, IL‐1β is primarily synthesized in and released from myeloid cells. Furthermore, to be active as a cytokine, it must undergo proteolytic processing by the active form of caspase 1 within an inflammasome complex. The mature IL‐1β, in turn, is released into the extracellular space via the gasdermin D pore complex and induces pyroptotic cell death. IL‐1β and IL‐1α bind to the IL1R1 receptor and thus activate the same signaling cascade. A further similarity between the two cytokines is that they both undergo proteolytic cleavage, but in contrast to IL‐1β, the pro‐form of IL‐1α is already biologically active. This activity can be further increased by proteolytic processing [13, 15]. IL‐1α, in contrast to IL‐1β, is broadly produced in a variety of cells. Its release during the process of uncontrolled cell death characterizes its role as an alarm signal triggering immune response activation in the event of severe cell damage. In the initiation of an episode of acute pancreatitis, uncontrolled cell death plays a pivotal role in determining severity and course of the disease. Only a small proportion of acinar cells undergo apoptosis; the majority of cells die by necrosis or necroptosis [26, 27]. In the present study, we demonstrate that damaged acinar cells express *Il1a* and *Il1b* at the mRNA level. This phenomenon can be attributed to the early activation of NFκB. In our experimental animal model, NFκB activation can be observed in pancreatic acinar cells within a few minutes after CCK stimulation [20]. The transcription factor NFκB positively regulates the transcription of various inflammatory mediators and proteins, such as cytokines and chemokines. Cytokines of the IL‐1 family are also under the control of NFκB [28]. We observed a significantly stronger transcriptional induction of *Il1a* in comparison to *Il1b*. Furthermore, no active caspase 1 was detected in acinar cells, nor was IL‐1β detected in the culture medium of CCK‐stimulated acinar cells. This was different for IL‐1α that is released from necrotic acinar cells at an early stage in a sterile inflammation like AP to induce an inflammatory cascade via the IL1R1 receptor.

In contrast to our initial hypothesis, we observed that fibroblasts rather than macrophages were activated by IL‐1α. Resident fibroblasts, or quiescent pancreatic stellate cells are present in healthy pancreatic tissue and synthesize the IL1R1 receptor [29, 30]. Upon stimulation with IL‐1α, fibroblasts differentiate into a pro‐inflammatory phenotype, a process that has previously been documented in the context of pancreatic cancer [31] and colorectal cancer [32]. IL‐1α induces the translocation of NFκB into the cell nucleus followed by the transcription of genes encoding various pro‐inflammatory mediators, including IL‐6, CCL2, CXCL5, and CXCL1 [33]. These pro‐inflammatory fibroblasts thus can recruit immune cells to the pancreas, and blockage of this IL‐1α pathway was shown to reduce fibroblast‐mediated chemokine release [32, 33]. The present study suggests that crosstalk between pancreatic fibroblasts and immune cells and macrophages clearly affects the immune response during AP. In *Il1a‐/‐* animals, significantly reduced levels of serum chemokines resulted in diminished migration of CCR2^+^ leukocytes into the inflamed pancreas. Thus, tissue‐resident fibroblasts function as sensors of initial acinar cell damage and initiate the inflammation and repair processes by chemokine release, which in turn recruits immune cells to the site of damage.

In patients, AP manifests as severe necrotizing pancreatitis in approximately 20% of cases. This disease is often fatal, with limited causal therapeutic options. As the initiation of an inflammatory cascade represents a critical factor in determining the disease course, an effective therapy has to attenuate the damage as well as an excessive immune response. Protease inhibitors, which prevent the activation of potentially damaging pancreatic enzymes, have been used to treat AP, but their efficiency was largely disappointing. Overall, treatment with protease inhibitors did not significantly reduce the mortality rate associated with AP [34]. It is important to note that the initial events of the disease, which include intra‐acinar protease activation and the induction of the immune response, in most cases occur before patient hospitalization. Our study showed that a “therapeutic” blockade of the IL1R1 signaling pathway is able to mitigate inflammation and tissue damage in a mouse model of AP. This might represent a promising target for the treatment of acute pancreatitis.

Another consequence of the IL‐1α stimulation is a priming effect on fibroblasts, which increases their response to fibrogenic signals and promotes fibrogenesis. IL‐1α‐activated fibroblasts did not exhibit a pro‐fibrotic phenotype; however, they upregulate fibrogenesis‐associated receptors, including TGBR3, FGFR2, IL4Ra, and IL13Ra [25, 30, 35, 36, 37]. TGFBR3 is a transmembrane proteoglycan, which serves as a co‐receptor for TGF‐β ligands and can modulate TGF‐β signaling in a context‐dependent manner. This priming of fibroblasts for extracellular matrix production occurs in parallel with pro‐inflammatory cell differentiation and the secretion of cytokines and chemokines. Interestingly, the co‐stimulation of fibroblasts with IL‐1α and TGF‐β resulted in significantly elevated *Col1a1* and *Col3a1* transcript levels. TGF‐β is a classical activator of fibroblasts and a pivotal driver of fibrosis [10, 11, 12]. Upon its release by alternatively activated macrophages (CD206^+^) [10], the priming of tissue‐resident fibroblasts occurs in the early phase of AP and likely initiates subsequent fibrosis. Similar to TGF‐β, also the IL‐4/IL‐13 has been demonstrated to act directly on fibroblasts and to induce the production of extracellular matrix proteins such as COL6A5 [25]. Besides TGFBR3 we observed an up‐regulation of the IL‐4 and IL‐13 receptors on pancreatic fibroblasts. Similar to the effect on TGF‐β‐signalling, we observed an enhanced transcription of collagens such as COL6A5, which suggests a general priming effect on fibroblast activation. Our finding indicates a direct regulatory connection of acinar cell necrosis and fibrogenesis. Interestingly, transgenic mice overexpressing human mature IL‐1β in the pancreas develop a chronic form of pancreatitis, characterized by severe inflammation and excessive pancreatic fibrosis [38]. Like IL‐1α, IL‐1β also binds to the IL‐1 receptor 1 and activates the same downstream signaling cascade [14, 15]. However, IL‐1β, in contrast to the more ubiquitous IL‐1α, is exclusively produced by myeloid cells like macrophages and secreted in response to inflammatory stimuli. Nevertheless, also IL‐1β has been demonstrated to significantly impact disease progression [7, 16, 17].

In recent years, it has been demonstrated that IL‐1α is a driving factor of tissue fibrosis in several other diseases. In a mouse model of bleomycin‐induced pulmonary fibrosis, it could be shown that both *Il1a‐/‐* mice as well as IL1R1‐deficient animals exhibited less fibrosis compared to wild‐type controls [22]. The authors demonstrated that IL‐1α is released in higher concentrations through endothelial damage than IL‐1β is released by macrophages. Therefore, the impact of IL‐1α seems more pronounced on fibrosis compared to IL‐1β, similar to our observations in the context of pancreatic fibrosis. It has also been shown that the IL‐1R1 signaling pathway plays an important role in the progression of liver fibrosis. IL1R1‐deficient mice exhibit reduced activation of hepatic stellate cells and show significantly lower production of extracellular matrix proteins, such as type I and III collagens [39]. Similarly, in renal fibrosis, it has been shown that the activation of fibroblasts via the IL‐1 signaling pathway induces tubular fibrosis [40]. Besides fibrotic diseases, in pancreatic cancer it has also been shown that the IL1R1 signaling plays a crucial role for fibroblast activation. For cancer‐associated fibroblasts (CAFs), the IL1R1 signaling pathway is essential for the differentiation of iCAFs [41]. It appears that the IL‐1 signaling pathway plays a crucial role for fibroblast function and regulates the immune response, fibrosis, and wound healing in multiple diseases.

Taken together, our results suggest that the IL1R1 signaling pathway could represent a suitable therapeutic target for the treatment of pancreatitis. First, its blockage has a dampening effect on the inflammatory cascade during AP, thereby counteracting hyperinflammation. Second, it reduces the priming of fibroblasts and prevents excessive fibrosis (Figure 8M). Anakinra is a human IL1 receptor antagonist approved for therapy and prevents the binding of IL‐1α and IL‐1β to the IL1 receptor [42]. We clearly show that Anakinra treatment is able to impede both pro‐inflammatory [43] and a pro‐fibrotic signaling pathways during pancreatitis. Similar observations have been reported for liver disease [44]. During AP, it is necessary to counteract the excessive pro‐inflammatory immune response to prevent the development of a systemic inflammatory response syndrome (SIRS). In AP Anakinra has an anti‐inflammatory effect, as it blocks the signaling pathways of both IL‐1α and IL‐1β. Our finding may have therapeutic implications for patients suffering from acute and chronic pancreatitis. Anakinra has already received approval for the treatment of various chronic diseases, including rheumatoid arthritis [45]. A clinical challenge in the management of AP is the transition from hyperinflammation to immune‐suppression during the progression of the disease [5, 7]. Blockage of pro‐inflammatory signaling pathways carries the risk of immune paralysis, potentially causing severe complications including infection of pancreatic necrosis with commensal bacteria from the intestine [2, 3]. However, in contrast to most other immunomodulatory antibody therapies, Anakinra exhibits a comparably short half‐life [46]. Unlike the cytokine‐blocking antibodies canakinumab or bermekimab, which remain effective over weeks, Anakinra has a short half‐life of only hours which allows rapid treatment discontinuation in case of signs of hypo‐inflammation.

Our study has several limitations. The results are based on animal experiments and cell culture experiments. Clinical data or data from human subjects were not included in the study. Both the dosage of anakinra and the treatment regimen may need further optimization to increase the effectiveness; however, our study shows that blocking the IL1R1 receptor represents a therapeutic option. Further studies—and, above all, clinical trials—will have to be conducted in the future in order to demonstrate a translational benefit for the patients, especially in the prevention of a disease progression from mild AP to severe necrotizing pancreatitis or other life‐threatening complications. In chronic disease, treatment with anakinra could help to slow down the fibrogenic tissue replacement and preserve organ function.

In summary, we demonstrated that pancreatic acinar cells are a source of IL‐1α but not IL‐1β. IL‐1α directly activates tissue‐resident fibroblasts, which in turn regulate the recruitment of immune cells via the release of cytokines and chemokines. Thus, IL‐1α signaling fuels inflammation and tissue damage. Furthermore, the priming of fibroblasts via the IL1R1‐signaling pathway enhances tissue fibrosis. Blockage of the IL1R1 signaling pathway by Anakinra targets both hyperinflammation and tissue fibrosis and therefore represents a promising therapeutic approach for treating patients with acute and chronic fibrosis.

## 4 Material and Methods

### 4.1 Ethical Statements

All experimental animal procedures were approved by the local animal care committee (Landesamt für Landwirtschaft, Lebensmittelsicherheit und Fischerei Mecklenburg‐Vorpommern, protocol number: 7221.3‐1‐035/22). All animal experiments were performed in accordance with the ARRIVE guidelines.

Human chronic pancreatitis tissue FFPE samples were collected during the ChroPac trial (trial registration number in the ISRCTN (International Standard Randomised Controlled Trial Number) database: ISRCTN38973832) [47]. No written consent has been obtained from the patients as there is no patient‐identifiable data included in this case report/series.

### 4.2 Animal Experiments

Mice were maintained in the central animal facility at University Medicine Greifswald, in accordance with German Animal Protection Law. A maximum of five mice were kept per cage under pathogen‐free conditions (SPF) in a humidity‐controlled environment. All mice had free access to food and water ad libitum. *Il1a*‐/‐ mice (C57BL/6J‐*Il1a* ^em2Lutzy^/Mmjax, strain code #067031‐JAX) and *Nlrp3‐/‐* mice (B6.129S6‐*Nlrp3* ^tm1Bhk^/J, strain code #021302) were purchased from *The Jackson Laboratory*, and C57Bl/6‐J mice were obtained from *Janvier Labs* (France) and used as controls.

AP was induced by 8 h i.p. injections of caerulein (50 µg/kg/bodyweight) (4030451, Bachem, Bubendorf, Swiss), and CP was induced by 6 h i.p. injections of caerulein (50 µg/kg/bodyweight), three times per week over a time period of 4 weeks, as previously described [11]. *Il1a‐/‐* and wild‐type mice were used at an age of 8‐ to 16‐weeks.

Blockade of the IL1 receptor signaling in the AP model was performed by a single i.p. injection of 10 mg/kg/body weight Anakinra (Swedish Orphan Biovitrum AB, Stockholm, Sweden) 1 h after the first caerulein injection. Blockade of the IL1 receptor in the CP model was performed always 1 h before the first caerulein injection, three times a week over the period of 4 weeks.

Mice were anaesthetized by an i.p. injection of a combination of ketamine and xylazine, and sacrificed eight or 24 h after the first injection of caerulein or 28 days after the start of the CP induction. Blood samples were collected retro‐orbitally during anesthesia, then the mice were euthanized by cervical dislocation and the pancreas tissue and lung were removed immediately for further analysis.

### 4.3 Antibodies

Anti‐α amylase (sc‐46657, Santa Cruz, Dallas, TX, USA), anti‐CCR2 (ab273050, Abcam, Cambridge, UK), anti‐CD68 (ABIN181836, Antibodies‐online, Aachen, Deutschland), anti‐CD206 (OASA05048, aviva‐sysbio, San Diego, CA, USA), anti‐NFkBp65 (#8242, Cell Signaling, Danvers, USA), anti‐αSMA (M0851, Agilent DAKO, Santa Clara, USA), anti‐Ki67 (IHC‐00375, Bethyl, Montgomery, USA), anti‐FGFR (9740S, Cell Signaling, Danvers, USA), anti‐IL‐1α (D4F3S, Cell Signaling, Danvers, USA), anti‐IL1R1 (NB110‐8541, Novus Biotechnology, Denver, US), anti‐IL‐4R (PA5‐103142, Invitrogen), anti‐FAP (ab218164 Abcam, Cambridge, UK), anti‐mouse‐Cy3 (dilution 1:200, 115‐165‐166, Jackson ImmunoResearch) anti‐mouse‐FITC (dilution 1:200, 115‐095‐146, Jackson ImmunoResearch), anti‐rabbit‐Cy3 (dilution 1:200, 111‐165‐144, Jackson ImmunoResearch), anti‐rabbit‐Cy5 (dilution 1:200, 111‐175‐144, Jackson ImmunoResearch), anti‐rat‐Cy3 (dilution 1:200, 112‐165‐062, Jackson ImmunoResearch).

The following Antibodies were used for flow cytometry analysis: anti‐CD192/CCR2‐FITC (dilution 1:50, 150608, BioLegend), anti‐CD11b‐PerCP/Cyanine5.5 (dilution 1:50, 101228, BioLegend). anti‐Ly‐6G‐APC (dilution 1:50, 127614, BioLegend), anti‐CD18‐PE (dilution 1:50, 130‐104‐050, Miltenyi Biotec), anti‐CD206‐Brilliant Violet 421 (dilution 1:50, 141717, BioLegend), anti‐CD45‐APC/Cyanine7 (dilution 1:50, 157618, BioLegend), anti‐CD124/IL‐4Rα‐PE/Cyanine7 (dilution 1:50, 144806, BioLegend), anti‐CD140a‐PE/Cyanine5 (dilution 1:50, 135920, BioLegend), anti‐alpha‐Smooth Muscle Actin‐Alexa Fluor 488 (dilution 1:50, 53‐9760‐82, Invitrogen), anti‐FAP‐Alexa Fluor 594 (dilution 1:50, FAB9727T, R&D Systems), anti‐alpha‐Smooth Muscle Actin‐Alexa Fluor 647 (dilution 1:50, 18701902, Novus Biologicals).

### 4.4 Culture of Bone Marrow‐Derived Macrophages (BMDM)

The isolation of bone marrow cells was conducted from the femur and tibia of C57BL/6‐J mice. The cells were subsequently cultured in RPMI medium supplemented with 10% FBS, 1% P/S, and 20 ng/mL murine M‐CSF (576406, BioLegend, San Diego, CA, USA) for a period of 7–10 days to induce the differentiation process of the macrophages.

### 4.5 Measurement of IL‐1β Release From BMDMs

BMDMs were stimulated for 2 h with 10 ng/mL LPS from *E. coli* (Sigma–Aldrich) as a priming signal, followed by stimulation with 1 µm nigericin (InvivoGen) for an additional 4 h or were co‐incubated with CCK‐stimulated acini for 6 h. Untreated BMDMs refer as negative control as well as CCK‐stimulated acini without co‐incubation with BMDMs. IL‐1β was determined in supernatant of cells by Mouse IL‐1β/IL‐1F2 Immunoassay (Biotechne / R&D Systems (Minneapolis, USA) Ref. P489015).

### 4.6 Isolation of Pancreatic Fibroblasts

Primary fibroblasts were isolated from the pancreas of C57BL/6‐J mice. Briefly, the pancreas was subjected to enzymatic digestion using the *Multi Tissue Dissociation Kit 3* (130‐110‐204, Miltenyi Biotec, Bergisch Gladbach, Germany) at 80 rpm, 37°C for 10 min. The tissue was dissociated by homogenisation with the *gentleMACS Dissociator* (130‐093‐235, Miltenyi Biotec) at 287 runs for 52 s in DMEM medium supplemented with 10% FBS, 1% P/S. The remaining acinar cells were removed by filtration through a 40 µm cell strainer and subsequent centrifugation (2300 rpm, 2:30 min). Pancreatic fibroblasts were cultivated in DMEM medium containing 10% FBS, 1% P/S, and 10 ng/mL FGF‐2 (130‐105‐786, Miltenyi Biotec) for a period of seven to 10 days. Cells were stimulated with 10 ng/mL recombinant mouse IL‐1α‐Protein (575002, BioLegend), 10 ng/mL recombinant Mouse IL‐4 Protein (#404‐ML, R&D Systems), 10 ng/mL recombinant Mouse IL‐13 Protein (#413‐ML, R&D Systems), and 10 ng/mL recombinant Mouse TGF‐β1 Protein (7666‐MB, R&D Systems).

### 4.7 Proliferation Assay

The proliferation of pancreatic fibroblasts was determined by using the *Cell Counting Kit 8* (CCK 8, GK10001, GlpBio, Montclair, CA, USA). Therefore, fibroblasts were seeded at a density of 1000 cells/well in 100 µL of culture medium in a 96‐well plate. The cells were then stimulated with 10 ng/mL of IL‐1α for 24 h. Subsequently, 10 µL of the CCK8 solution was added, and the cells were incubated for 2 h at 37°C. The absorption was measured at a wavelength of 450 nm.

### 4.8 Isolation of Pancreatic Acini

Acinar cells were isolated from the murine pancreas of wild type and *Il1a‐/‐* mice. Pancreatic tissue was minced into small pieces and subjected to enzymatic digestion by collagenase digestion (Collagenase of *Clostridium histolyticum* (EC.3.4.24.3) from Serva, Heidelberg, Germany) [18]. Cells were cultivated and stimulated at various time points with 0.001 mm Cholecystokinin (CCK), CCK‐Octapeptid (J66669.EXD Thermo‐Fisher Scientific, Bremen, Germany) in Dulbecco's modified Eagle's medium, which was supplemented with 10 mm HEPES, 2% BSA, and PenStrep.

### 4.9 Protease Activity Measurement

Acinar cells were collected in PBS, washed, and then homogenised using ultrasound. Protease activity was measured in the cell homogenate using fluorogenic substrates in kinetics over 1 h at 37°C. Trypsin activity was measured in 100 mm TRIS, 5 mm CaCl2 at pH 8.0 using 10 µm of the substrate Ile‐Pro‐Arg‐R110 (R6505, ThermoFisher Scientific, Waltham, MA, USA). Caspase‐1 activity was measured using 10 µm of the substrate VI Ac‐Tyr‐Val‐Ala‐Asp‐AFC (CAS 219137‐85‐6, Calbiochem Merck, Darmstadt, Germany) in PBS; caspase‐3 activity was measured using 20 µm of the substrate R110‐Ile‐Glu‐Thr‐Asp (R110‐DEVD) from AAT Bioquest (Cat. No. 13430) in PBS; and calpain activity was measured using 20 µm of the substrate Suc‐Leu‐Tyr‐AMC (ref: 208731, Calbiochem Merck) in PBS.

Acinar cell PI assay was performed in buffer containing 24.5 mm HEPES, 96 mm NaCl, 11.5 mm glucose, 6 mm KCl, 1 mm MgCl_2_ 6H_2_O, 0.5 mm CaCl_2_ 2H_2_O, 2.5 mm NaH_2_PO_4_ H_2_O, 5 mm sodium fumarate, 5 mm sodium glutamate, 5 mm sodium pyruvate, 1% BSA, DMEM and propidium iodide. PI uptake was measured by a SpectraMax i3x, acinar cells were quantified by a MiniMax imaging cytometer (Molecular Devices).

### 4.10 Cytokine and Chemokine Measurements in Serum and Supernatant

Levels of cytokines and chemokines were measured in cell culture media of stimulated acinar cells and fibroblasts, as well as in serum samples. The following commercially available kits were used according to the manufacturers' protocols: *Mouse IL‐6 ELISA Kit* (M6000B‐1, bio‐techne, Minneapolis, MN, USA), *Cytometric Bead Array (CBA) Mouse inflammation kit* (552364, BD Bioscience, San Jose, CA, USA), *LEGEDplex Custom Mouse Panel 744* (BioLegend, San Diego, CA, USA) and *LEGENDplex MU Proinflam. Chemokine Panel* (741295, BioLegend).

### 4.11 Flow Cytometry

As previously described, pancreas and blood samples were processed into single‐cell suspension and subsequently stained for surface markers [11, 48]. In order to block non‐specific binding of antibodies during the labeling procedures, *FcR Blocking Reagent* (130‐092‐575, Miltenyi Biotec) was used. The labeling of chemokine receptors was conducted over a period of 15 min at 37°C. Intracellular marker labeling was performed following permeabilization of cell membranes with the *Transcription Factor Staining Buffer Set* (130‐122‐981, Miltenyi Biotec). Antibodies were diluted in staining buffer (PBS, 2 % FCS, 0.02 % sodium azide, 2 mm EDTA). Data acquisition was performed using LSR II (BD Bioscience), and data analysis was conducted using FlowJo software version 10 (BD Bioscience).

### 4.12 Measurement of Myeloperoxidase Activity

Lung tissue was homogenized in 20 mm potassium phosphate buffer (pH 7.4). The tissue homogenate was centrifuged, and the pellet was resuspended in 50 mm potassium phosphate buffer solution (pH 6) containing 0.5% cetyltrimethylammoniumbromide. Four freezing/thawing cycles were performed to extract MPO. The measurement of myeloperoxidase activity was performed in a 50 mm potassium phosphate buffer solution (pH 6) containing 0.53 mm O‐dianisidine and 0.15 mm H_2_O_2_. MPO activity was measured kinetically at 30°C for 10 min in a Spectrophotometer (Spectramax, Molecular Devices, San Jose, CA, USA) as previously described [8]. The enzyme activity was measured against a purified MPO standard (Cat# 475911, Calbiochem) and adjusted to the protein content of the samples.

### 4.13 Amylase Lipase

Activity of serum amylase was measured by colorimetric assay (AMYL2 Ref: 03183742122, Roche Hitachi). Lipase activity in serum was also measured by colorimetric assay (LIPC Ref: 03029590322, Roche Hitachi).

### 4.14 Measurement of Fecal Elastase and Chymotrypsin Activity

Fecal elastase and chymotrypsin activities were measured in stool samples from CP and control animals. Samples were resuspended and sonicated in 500 mmol/L NaCl, 100 mmol/L CaCl_2_ , containing 0.1% Triton X‐100. Elastase activity was measured by fluorometric enzyme kinetics over 1 h at 37°C by the usage of 0.12 mm elastase substrate (CBZ‐Ala‐Ala‐Ala‐Ala)2‐R110 (R6506, ThermoFisher Scientific). Chymotrypsin activity was also measured as enzyme kinetics over 1 h at 37°C by the usage of 0.12 mm substrate Suc‐Ala‐Ala‐Pro‐Phe‐AMC (CAS Number: 71973‐79‐0, Echelon Biosciences). All kinetics were measured in 100 mmol/l Tris buffer containing 5 mmol/L CaCl2 at pH 8.0. Data were normalized to sample weight.

### 4.15 Histology

Histological analysis was conducted on 2‐µm‐thick sections of FFPE tissue and fresh frozen OCT‐embedded tissue. Immunostaining of FFPE samples was performed subsequent to deparaffinization, rehydration, and antigen retrieval steps. Cryosections were fixed with acetone. Sections were subjected to blocking (0.02% Tween20 and 10% FCS in 1X PBS) and then incubated in primary antibodies (1:200 in blocking buffer at 4°C overnight). This initial incubation was then followed by an additional round of incubation with appropriate secondary antibodies (1:200) for 1 h at room temperature. The tissue sections were counterstained with DAPI (6335.1, Carl ROTH, Karlsruhe, Germany), or DAB chromogens (SK‐4100, Vector Laboratories, Newark, CA, USA) were applied, followed by hematoxylin counterstain.

Immunofluorescence labeling of fibroblasts was performed in chamber slides. Cells were stimulated with 10 ng/mL IL‐1α for 24 h and subsequently washed carefully with PBS before being fixed in ice‐cold acetone for 10 min. Cells were subjected to a blocking and permeabilization process, involving the use of 0.02% Tween 20 and 10% FCS in 1X PBS. Antibodies at a 1:200 dilution were employed for overnight incubation at 4°C. Secondary antibody incubation was then conducted for 1 h at room temperature. The nuclei were stained with DAPI, and the slides were mounted using DACO mounting medium for fluorescence slides.

Hematoxylin and eosin (12156, Morphisto, Offenbach am Main, Germany) and Azan staining (12079, Morphisto) were performed on 2‐µm sections of FFPE pancreas and lung tissues. A blinded scoring system was employed to grade the tissue damage, with the assessment of necrosis, edema, and infiltration (0 = absent, 1 = minor, 2 = present, 3 = abundant).

The imaging process was conducted using a fluorescence microscope (Olympus IX81, Olympus, Waltham, MA, USA) or a slide scanner (Pannoramic MIDI II platform, Sysmex, Kobe, Japan) and subsequently processed using QuantCentre software (Sysmex).

### 4.16 Reverse Transcription Quantitative PCR (RT‐qPCR)

Total RNA was isolated from acinar cells, fibroblasts, and macrophages using Trizol (155966018, Invitrogen, Waltham, MA, USA) and the RNA concentration was determined using the *Eppendorf BioPhotometer* (Eppendorf, Hamburg, Germany). Single‐strand cDNA was synthesised using the *High‐Capacity cDNA Reverse Transcription Kit* (4374967, Thermo Fisher Scientific). SYBR Green PCR Master Mix (4309155, Thermo Fisher Scientific) was used to perform quantitative PCR in triplicate using the QuantStudio 6 Flex Real‐Time PCR System (Thermo Fisher Scientific). Gene expression in terms of mRNA levels was normalized to the calibrator gene *RNs5* and calculated using the ΔΔCt method.The following oligonucleotide primers used in this study were purchased from Invitrogen (Waltham, MA, USA): Rn5s forward 5′‐GCCCGATCTCGTCTGATCTC‐3′ reverse 5′‐GCCTACAGCACCCGGTATTC‐3′, Actb forward 5′‐GAGGTATCCTGACCCTGAAGTA‐3′ reverse 5′‐CACACGCAGCTCATTGTAGA‐3′, Acta2 forward 5′‐GCCAGTCGCTGTCAGGAACCC‐3′ reverse 5′‐CCAGCGAAGCCGGCCTTACA‐3′, Cd40 forward 5′‐GAGTCAGACTAATGTCATCTGTGGTT‐3′ reverse 5′‐ACCCCGAAAATGGTGATG‐3′, Cd80 forward 5′‐ CGCAACCACACCATTAAGTG‐3′ reverse 5′‐GACGACTGTTATTACTGCGCC‐3′, Cd86 forward 5′‐ACGATGGACCCCAGATGCACCA‐3′ reverse 5′‐GCGTCTCCACGGAAACAGCA‐3′, Col1a1 forward 5′‐CAGACTGGCAACCTCAAGAA‐3′ reverse 5′‐CAAGGGTGCTGTAGGTGAAG‐3′, Col3a1 forward 5′‐ ATGGCTCACCAGGACAAAG‐3′ reverse 5′‐CACCAGGACTGCCGTTATT‐3′, Col6a5 forward 5′‐GCGCCAACCAGTCTGAATTC‐3′ reverse 5′‐TCCTCATCTGCTCAATGGCG‐3′, Ccr2 forward 5′‐CAAGAGCTTGATGAAGGGGC‐3′ reverse 5′‐ATGACCAACATGTTGCCCAC‐3′, Ccr5 forward 5′‐GCTGCCTAAACCCTGTCATC‐3′ reverse 5′‐GTTCTCCTGTGGATCGGGTA‐3′, Fn1 forward 5′‐ GCCTGAGGTGGACCCCGCTA‐3′ reverse 5′‐GGGCCCAAGTGACCCGCATC‐3′, Il1a forward 5′‐TCTCAGATTCACAACTGTTCGTG‐3′ reverse 5′‐AGAAAATGAGGTCGGTCTCACTA‐3′, Il1b forward 5′‐GAGGACATGAGCACCTTCTTT‐3′ reverse 5′‐GCCTGTAGTGCAGTTGTCTAA‐3′, ′, Il6 forward 5′‐ CCAGAGTCCTTCAGAGAGATACA‐3′ reverse 5′‐CCTTCTGTGACTCCAGCTTATC‐3′, Il13ra1 forward 5′‐ACCTGTGACGAATTTGAGCG‐3′ reverse 5′‐CGATGAGTTTCTGGAGCAAT‐3′, Pdgfrb forward 5′‐GCGAGGTGGACTTCCTGGAG‐3′ reverse 5′‐GCGGTCCCAGGAGCCATAAC‐3′, Sell forward 5′‐GTAGCCGTCATGGTCACCGC‐3′ reverse 5′‐TAATGTGGGAGATGCCTGCG‐3, Tgfbr3 forward 5′‐CCCTGCATCTGAACCCCATT‐3′ reverse 5′‐ACCACAGAACCCTCCGAAAC‐3′.

### 4.17 Microarray‐Based Transcriptome Analysis of BMDM and Pancreatic Fibroblasts After IL‐1α Stimulation

Three biological replicates of bone marrow‐derived macrophages from wild‐type mice and three replicates of pancreatic fibroblasts were either stimulated with IL‐1α [10 ng/mL] or left unstimulated (control condition) for 24 h, respectively. Cells were lysed using 1 mL TRIzol, and lysates were snap‐frozen in liquid nitrogen and stored at (−70°C) until RNA extraction. Total RNA was isolated following the manufacturer's instructions for total RNA isolation with TRIzol (Thermo Fisher Scientific, Waltham, MA, USA). The upper aqueous RNA‐containing phase was further processed according to the protocol for RNA Clean‐up and Concentration from Phenol/Guanidine‐based RNA isolation (RNA_Clean‐Up and Concentration Kit, NorgenBiotek, Thorold, Canada). After purification and quality assessment of the total RNA preparations using an Agilent Bioanalyzer (average RNA‐integrity number BMDM RIN = 10, RNA concentration: 223 ± 16 ng/µL, fibroblasts RIN = 10, RNA concentration 145 ± 19 ng/µl), transcriptional profiling of stimulated and unstimulated macrophages and pancreatic fibroblasts derived from wild type mice was performed using the WT Plus Kit and Clariom S mouse arrays (Thermo Fisher Scientific, Waltham, MA, USA) according to the manufacturer's instructions.

For probe set extraction and normalization, raw expression data were transferred to the Transcriptome Analysis Console (TAC, 4.0.2.15, Thermo Fisher Scientific, Waltham, Massachusetts, USA). The generated gene expression data sets were submitted to Gene Expression Omnibus (GEO). Statistical analysis of the case‐control study based on three biological replicates for each group was performed using analysis of variance (ANOVA) with e‐Bayesian correction. Statistically significant differential gene expression in terms of transcript levels was defined as a fold change of > |1.5| between the compared conditions and an FDR < 0.05. Microarray data have been deposited in the National Center for Biotechnology Information GEO database and are accessible through the following GEO accession number: GSE310835.

### 4.18 Proteome Analysis of BMDM and Pancreatic Fibroblasts After IL‐1α Stimulation

Fibroblasts or BMDM were harvested in Trizol and treated according to the manufacturer's protocol. Total protein was obtained from the lower organic phase by isopropanol precipitation. Protein pellets were reconstituted in Tris‐HCl (10 mm, pH 7.5) with 5% SDS. Protein concentrations were determined by BCA assay (Pierce/Thermo Fisher Scientific). Further sample preparation and mass spectrometric analysis were carried out as described earlier [49]. In summary, protein lysates were subjected to an adapted bead‐based SP3 protocol [50]. Four micrograms of each protein sample were reduced by dithiothreitol (2.5 mm, 30 min at 37°C) and alkylated with iodoacetic acid (10 mm, 30 min at 37°C in the dark). Following this, proteins were digested by LysC/trypsin (Promega, Walldorf, Germany) in a protein to enzyme ratio of 25:1 overnight at 37°C. Peptides were separated by LC (Ultimate 3000, Thermo Electron, Bremen, Germany) prior to data‐independent acquisition of mass spectra on a QExactive Plus mass spectrometer (Thermo Electron). The analysis of MS data was conducted utilizing the DirectDIA algorithm implemented in Spectronaut (v18.3, Biognosys, Zurich, Switzerland), employing an Uniprot database (version 2022_4) that was restricted to *Mus musculus* (*n* = 17 107). Carbamidomethylation at cysteine was designated as a static modification, while oxidation at methionine and protein N‐terminal acetylation were defined as variable modifications. Additionally, up to two missed cleavages were permitted. The presence of two or more unique+ razor peptides, which were identified and quantified per protein, was a prerequisite for proteins to be considered for further analysis. Peptides with oxidized methionine were excluded. Data were median‐normalized at the ion level prior to statistical analysis using the algorithm ROPECA [51]. The application of a reproducibility‐optimised test statistic (utilising the ROTS package) facilitated the identification of binary differences. Only proteins with adjusted p‐value < 0.05 and a fold change of >|1.3| were considered as significantly differentially abundant. A detailed description of data acquisition and search parameters is provided in Tables S1 and S2.

### 4.19 Data Analysis and Statistics

Statistical analyses were performed using GraphPad Prism (version 5.0.0 or later) with the statistical tests indicated in the individual figure legends. The data are presented as mean ± standard error of the mean (SEM), as indicated in the corresponding figure legends. For all experiments involving two groups, two‐tailed unpaired *t*‐tests were employed. For experiments with more than two groups that were normally distributed, a one‐way ANOVA test was performed. For samples that were non‐normally distributed, the Mann–Whitney or Kruskal–Wallis tests were utilised. P‐values <0.05 were considered statistically significant. The final graphing and figure design were performed using *R* and *PowerPoint* (Office 2019 or later).

## Author Contributions

Concept of the study: M.S., F.U.W., and J.G. Data acquisition and interpretation: H.M., S.B., H.S., S.A., L.S., B.L. Becker, E.H., G.H., U.V., B.M. Bröker, L.Z., F.U.W., M.S., and J.G. Writing committee: M.S., F.U.W., and J.G. Correction of manuscript and approval of final version: all.

## Funding

This work was supported by Deutsche Forschungsgemeinschaft (DFG SE 2702/2‐3, DFG SE 2702/4‐1 (DFG project number: 576085219), GL 1096/1‐1, ZI 2071/2‐1, GRK 2719 A1 and C1 (DFG project number: 443535983), RTG 2901 SYLOBIO project E02 (DFG project number: 501988175)).

## Conflicts of Interest

The authors declare no conflicts of interest.

## Supporting information




**Supporting File**: advs77558‐sup‐0001‐SuppMat.docx.

## Data Availability

The data that support the findings of this study are openly available in Gene Expression Omnibus at https://www.ncbi.nlm.nih.gov/geo/, reference number GSE310835.
