# Supplementary material for: Interleukin‐1α Mediates Pancreatic Fibroblast Activation, Regulates Immune Cell Recruitment and Fibrosis in Acute and Chronic Pancreatitis
Source: Adv Sci (Weinh). 2026 Sep 3:e77558. Online ahead of print. doi: 10.1002/advs.77558 (PMC13542390; doi:10.1002/advs.77558)
Supplement: Supplementary file 1 [file ADVS-9999-e77558-s001.docx]

Supplementary Figure 1:

**Supplementary Figure 1:** Effect of IL-1α treatment (10ng/ml) on gene expression of pancreatic fibroblasts as determined by microarray-based transcriptome analyses (**A**) The most affected Ingenuity Canonical Pathways. Pathways predicted to be downregulated with a z-score < -2 are shown in blue, while up-regulated pathways with a z-score > +2 are shown in red, pathways with no available z-score are shown in grey. (**B**) Volcano plots illustrating affected up-stream regulators after treatment. The Volcano plots reflect the different categories of upstream regulators (complex, transcription factors, kinases and phosphatases, enzymes and peptidases, cytokines and growth factors, G-protein coupled receptors, and transmembrane receptors, group and other). (**C**) Immunofluorescent labelling of Ki67 in isolated pancreatic fibroblasts +/- 10 ng/ml IL-1α for 24 hours. Bar graph illustrates increased cell viability by CCK8 assay (n=5). Differences were tested for statistical significance by unpaired student’s t-test for independent samples and significance levels of p<0.05 are marked by an asterisk.

Supplementary Figure 2:

**Supplementary Figure 2:** Impact of stimulation of cells isolated from wild type mice with 10 ng/ml recombinant murine IL-1α for 24 hours on the protein pattern as determined by proteome analysis. (**A**) Protein level alterations observed in bone marrow derived macrophages (BMDMs). Bar graph and Volcano plot illustrate differentially abundant proteins in BMDMs (n=3). (**B**) Protein level alterations observed in fibroblasts isolated from the pancreas. Bar graph and Volcano plot illustrate differentially abundant proteins in fibroblasts (n=3). Significance was tested using ROPECA.

Supplementary Figure 3:

**Supplementary Figure 3:** H&E histology of pancreatic tissue was analysed by histology scoring from wild type and *Il1a*-/- mice after induction of acute pancreatitis by caerulein (n=10). (**A**) Infiltration of immune cells, (**B**) necrosis of acinar cells and (**C**) pancreatic oedema were scored. (**D**) Measurement of lipase activity in serum of mice. Differences were tested for statistical significance by unpaired student’s t-test for independent samples and significance levels of p<0.05 are marked by an asterisk.

Supplementary Figure 4:

**Supplementary Figure 4:** (**A**) Scatter plots of flow cytometry analyses of blood leukocytes from wild type and *Il1a*-/- mice during acute pancreatitis to distinguish CD11b^+^/LY6G^-^ monocytes from CD11b^+^/LY6G^+^ neutrophils from wild type and *Il1a-/-* mice during acute pancreatitis. (**B**) Scatter plots of flow cytometry analyses of blood leukocytes from wild type and *Il1a-/-* mice during acute pancreatitis to identify CD4^+^ T helper cell population.

Supplementary Figure 5:

**Supplementary Figure 5:** H&E histology of pancreatic tissue was analysed by histology scoring from PBS and Anakinra treated mice after induction of acute pancreatitis by caerulein (n=10). (**A**) Infiltration of immune cells, (**B**) necrosis of acinar cells and (**C**) pancreatic oedema were scored. (**D**) Measurement of lipase activity in serum of mice. Differences were tested for statistical significance by unpaired student’s t-test for independent samples and significance levels of p<0.05 are marked by an asterisk.

Supplementary Figure 6:

**Supplementary Figure 6:** Immunofluorescent co-labeling of αSMA and the IL1R1 in human chronic pancreatitis tissue.

Supplementary Figure 7:

**Supplemental Figure 7:** The genetic deletion of NLRP3 has no impact on CP. (**A**) Measurement of cytokine levels of IL-1α and IL-1β in the serum of untreated control animals, wild-type animals with CP, *Il1a-/-* animals with CP, and wild-type animals with CP receiving anakinra treatment (n=10 each group). Differences were tested for statistical significance by Kruskal-Wallis-test followed by Dunn's multiple comparisons test, significance levels of p<0.05 are marked by an asterisk. (**B**) Chronic pancreatitis was induced by repetitive caerulein injections over four weeks in wild type and *Nlrp3-/-*mice (n=10 each group). (**C and D**) Azan blue stained CP tissue sections illustrate the tissue fibrosis. A quantification of the stained area is shown in the bar chart. (**E**) Immunofluorescent labeling of fibroblasts in CP tissue sections of mice by αSMA (green) and FGFR (red). The quantification of FGFR^+^ cells is shown in the bar graph. (**F**) Immunofluorescent labeling of CD206^+^ macrophages and CCR2^+^ immune cells in CP tissue sections of mice and a quantification of these cells. Differences were tested for statistical significance by unpaired student’s t-test for independent samples and significance levels of p<0.05 are marked by an asterisk.

**Supplemental Table 1**: A LC-MS/MS parameter (data independent mode; quantitative data)

| **Data independent analyses (DIA)** | |  |
| --- | --- | --- |
| **reversed phase liquid chromatography** | **Ultimate 3000 RSLC (Thermo Scientific)** | |
| Trap column | 75 μm inner diameter, packed with 3 μm C18 particles (Acclaim PepMap100, Thermo Scientific) | |
| Analytical column | 75 μm inner diameter, packed with 2.6 μm C18 particles (Accucore, 25 cm, Thermo Scientific) | |
| Flow rate | 300 nl/min | |
| column oven temperature | 40°C | |
| buffer system | binary buffer system consisting of 0.1% acetic acid in HPLC-grade water (buffer A) and 100% ACN in 0.1% acetic acid (buffer B) | |
| gradient | gradient of buffer B: 2min 2% to 5 %, 8min 5%, 120min 5% to 25%, 5min 25 to 40%, 2 min 40% to 90%, 5 min 90%, 1 min 90% to 2%, 7 min 2% | |
| **Mass spectrometer** | Q Exactive Plus mass spectrometer (Thermo Scientific) | |
| operation mode | data-independent | |
| electrospray | Nanospray Flex Ion Source | |
| **Full MS** |  | |
| MS scan resolution | 70,000 | |
| Full MS AGC target | 5.00E+06 | |
| maximum ion injection time for the MS scan | 120 ms | |
| Scan range | 350 to 1650 *m/z* | |
| Spectra data type | profile | |
| **dd-MS2** |  | |
| Resolution | 35,000 | |
| MS/MS AGC target | 3.00E+06 | |
| maximum ion injection time for the MS/MS scans | auto | |
| Spectra data type | profile | |
| selection for MS/MS | 1 | |
| isolation window | 19 windows *m/z* 24-222 | |
| Fixed first mass | 200 | |
| dissociation mode | higher energy collisional dissociation (HCD) | |
| normalized collision energy | fixed, 27.5% | |
| dissociation mode | HCD | |

**Supplemental Table 2**: Spectronaut parameters for peptide/Protein identification and intensity extraction

| Spectronaut 18.3.230830.5060 |  |
| --- | --- |
| Computer Name: AGVOE-SPECTRONA |  |
| User Domain Name: AGVOE-SPECTRONA |  |
| User Name: spectronaut |  |
| Analysis Mode: UI |  |
| Analysis Type: directDIA |  |
|  |  |
| Settings Used: |  |
| **Pulsar Search\Peptides** |  |
| Toggle N-terminal M: | True |
| Min Peptide Length: | 7 |
| Max Peptide Length: | 52 |
| Missed Cleavages: | 2 |
| Digest Type: | Specific |
| Enzymes / Cleavage Rules: | Trypsin/P |
| **DIA Analysis\Data Extraction** |  |
| MS1 Mass Tolerance Strategy: | Dynamic |
| Correction Factor: | 1 |
| MS2 Mass Tolerance Strategy: | Dynamic |
| Correction Factor: | 1 |
| Intensity Extraction MS1: | Maximum Intensity |
| Intensity Extraction MS2: | Maximum Intensity |
| **DIA Analysis\XIC Extraction** |  |
| XIC IM Extraction Window: | Dynamic |
| Correction Factor: | 1 |
| XIC RT Extraction Window: | Dynamic |
| Correction Factor: | 1 |
| **Pulsar Search\Modifications** |  |
| Max Variable Modifications: | 5 |
| **Database** |  |
| Original File: | Uniprot_04_2022.fasta (*Mus musculus*) |
| **Select Modifications:** |  |
| Fixed Modifications:: | Carbamidomethyl (C) |
| Variable Modifications: : | Acetyl (Protein N-term), Oxidation (M) |
| **DIA Analysis\Calibration** |  |
| MS1 Mass Tolerance Strategy: | System Default |
| MS2 Mass Tolerance Strategy: | System Default |
| **DIA Analysis\Identification** |  |
| Machine Learning: | Per Run |
| Protein Qvalue Cutoff (Experiment): | 0.01 |
| Protein Qvalue Cutoff (Run): | 0.05 |
| Exclude Single Hit Proteins: | False |
| PTM localization | True |
| Probability cut-off | 0.75 |
| Pvalue Estimator: | Kernel Density Estimator |
| Precursor Qvalue Cutoff: | 0.001 |
| Single Hit Definition: | By Stripped Sequence |
| **DIA Analysis\Quantification** |  |
| Amino acids | False |
| Interference Correction: | True |
| MS1 Min: | 2 |
| MS2 Min: | 3 |
| Fragment ions: |  |
| Best N fragments per peptide | True |
| Min | 6 |
| Max | 10 |
| Ion Charge | False |
| Ion Type | False |
| Protein LFQ Method: | Automatic |
| Major (Protein) Grouping: | by Protein Group Id |
| Minor (Peptide) Grouping: | by Stripped Sequence |
| Modifications | None |
| Minor Group Top N: | False |
| Minor Group Quantity: | Sum precursor quantity |
| Major Group Top N: | True |
| Min: | 2 |
| Max: | 3 |
| Major Group Quantity: | Mean peptide quantity |
| Quantity MS-Level: | MS2 |
| Quantity Type: | Area |
| Proteotypicity Filter: | Only Protein Group Specific |
| Data Filtering: | Identified (Qvalue) |
| Imputing Strategy: | Use Background Signal |
| Cross Run Normalization: | TRUE |
| Normalization Strategy | Local Normalization |
| **DIA Analysis\Workflow** |  |
| MS2 DeMultiplexing: | False |
| Run Limit for directDIA Library: | -1 |
| Method Evaluation: | False |
| Profiling Strategy: | iRT Profiling |
| Profiling Row Selection: | Minimum Qvalue Row Selection |
| Qvalue Threshold: | 0.001 |
| Profiling Target Selection: | Profile only non-identfied Precursor |
| Identification Criterion: | Qvalue |
| Threshold: | 0.001 |
| Carry-over exact Peak Boundaries: | False |
| Unify Peptide Peaks Strategy: | Select corresponding Peak |
| **DIA Analysis\Post Analysis** |  |
| Calculate Sample Correlation Matrix: | True |
| Calculate Explained TIC: | None |
| Differential Abundance Grouping: | Major Group (Quantification Settings) |
| Smallest Quantitative Unit: | Precursor Ion (Quantification Settings) |
| Use All MS-Level Quantities: | False |
| **DIA Analysis\Pipeline Mode** |  |
| Post Analysis Reports: |  |
| Scoring Histograms: | True |
| Data Completeness Bar Chart: | True |
| Run Identifications Bar Chart: | True |
| CV Density Line Chart: | True |
| CVs Below X Bar Chart: | True |
| Generate SNE File: | True |
| Store Iontraces in SNE: | False |
| Report Schema: | C_FunGene_complex (Normal) |
| Reporting Unit: | Across Experiment |
